# Self-reported bovine milk intake is associated with oral microbiota composition

**DOI:** 10.1371/journal.pone.0193504

**Published:** 2018-03-21

**Authors:** Ingegerd Johansson, Anders Esberg, Linda Eriksson, Simon Haworth, Pernilla Lif Holgerson

**Affiliations:** 1 Department of Odontology, Section of Cariology, Umeå University, Umeå, Sweden; 2 Department of Odontology, Section of Paedodontics, Umeå University, Umeå, Sweden; 3 Medical Research Council Integrative Epidemiology Unit, Department of Population Health Sciences, Bristol Medical School, University of Bristol, Bristol, United Kingdom; 4 Bristol Dental School, University of Bristol, Bristol, United Kingdom; University of Washington, UNITED STATES

## Abstract

Bovine milk intake has been associated with various disease outcomes, with modulation of the gastro-intestinal microbiome being suggested as one potential mechanism. The aim of the present study was to explore the oral microbiota in relation to variation in self-reported milk intake. Saliva and tooth biofilm microbiota was characterized by 16S rDNA sequencing, PCR and cultivation in 154 Swedish adolescents, and information on diet and other lifestyle markers were obtained from a questionnaire, and dental caries from clinical examination. A replication cohort of 31,571 adults with similar information on diet intake, other lifestyle markers and caries was also studied. Multivariate partial least squares (PLS) modelling separated adolescents with low milk intake (lowest tertile with <0.4 servings/day) apart from those with high intake of milk (≥3.7 servings/day) based on saliva and tooth biofilm, respectively. Taxa in several genera contributed to this separation, and milk intake was inversely associated with the caries causing *Streptococcus mutans* in saliva and tooth biofilm samples by sequencing, PCR and cultivation. Despite the difference in *S*. *mutans* colonization, caries prevalence did not differ between milk consumption groups in the adolescents or the adults in the replication cohort, which may reflect that a significant positive association between intake of milk and sweet products was present in both the study and replication group. It was concluded that high milk intake correlates with different oral microbiota and it is hypothesized that milk may confer similar effects in the gut. The study also illustrated that reduction of one single disease associated bacterial species, such as *S*. *mutans* by milk intake, may modulate but not prevent development of complex diseases, such as caries, due to adverse effects from other causal factors, such as sugar intake in the present study.

## Introduction

Milk and dairy products are important sources of energy and macro- and micronutrients, but intake varies between and within populations due to traditions and ability to digest lactose [1]. Sweden is a country with a high prevalence of lactose tolerance [2] and has among the highest intake of bovine milk in the world [3, 4].

Bovine milk intake has been studied in relation to various diseases, including dental diseases, cardiovascular diseases, type 2 diabetes, and cancer [5–7]. Three independent studies from Sweden have recently reported increased mortality risk among subjects with the highest intake of non-fermented bovine milk [8, 9, 10]. Causality remains unclear but lactose exposure and modulation of microbiota have been suggested as potential mechanisms [8, 9]. The microorganisms in the gastro-intestinal (GI) tract are organised in habitat specific, resilient communities regulated by habitat and exposure influences, including pH, oxygen, nutrients, neighbouring species, anti- and probiotics and transmission and availability of attachment sites [11, 12]. In addition to nutritional components, human and bovine milk contain, a large number of bioactive proteins, glycoproteins, peptides and glycolipids with effects on inflammatory responses and cell signalling in the host and metabolism and adhesion of microorganisms in the GI canal [11]. Human milk (breastfeeding) is known to affect establishment of the gut [13,14] and oral [15, 16] microbiota of the infant. Similarly, bovine milk and milk components have been shown to affect the gut microbiome in animals [17, 18], and reduce adhesion and metabolism of the tooth colonizing, caries causing *Steptococcus mutans* in vitro [19–21]. Such effects have not been evaluated in vivo in man, but it may be hypothesized that bovine milk modulates the microbiota in the mouth and other parts of the GI tract in later child- and adulthood. The primary aim of the present study was to explore the oral microbiota by DNA based characterization and culturing in relation to variations in milk intake, and, following our findings, a secondary aim was to evaluate caries status by milk intake and potential confounding factors. We found that milk intake levels were associated with distinct microbiota in saliva and tooth biofilms with strikingly less cariogenic *S*. *mutans* by increasing milk intake but found no strong association with caries status, a finding potentially confounded by sugar intake.

## Subjects and methods

### Study group

The study group involved 17-year-old adolescents (n = 154), who were recruited from public dental health care clinics in the city of Umeå, Sweden. The study group has been described previously [22]. Adolescents who consented to participate, were healthy, had not taken antibiotics during the latest 6 months, and were able to communicate in Swedish or English. Study participants abstained from oral hygiene on the morning of their visit to the dental clinic. Whole chewing stimulated saliva was collected for 3 minutes, and supragingival biofilm was collected from all accessible tooth surfaces using sterile wooden toothpicks. Samples from the different tooth surfaces were pooled for the subject into 100 μl of TE buffer (10 mM Tris, 1 mM EDTA, pH 7.6). All samples were stored at -80°C.

### Replication cohort

To evaluate and possibly replicate potential findings from the study group, we included participants in the Gene-Lifestyle Interaction in Dental endpoints (GLIDE) database with information on caries prevalence and lifestyle variables from participating in a health screening program, the Västerbotten Intervention Program (VIP) [23]. VIP is a population-based health screening and intervention program which includes anthropometric measures and questionnaire information on diet, tobacco use and education. The average recruitment rate has been around 60% of potential participants, and no indication of a systematic selection bias has been found [24, 25]. Participants aged 30–64 years at time of participation in VIP, who had completed diet questionnaires to an adequate level and had data on caries status were eligible (n = 43,788). Of these participants, those with diet information 0 to 5 years before the dental examination were included in analysis (n = 31,571).

### Information on diet and other lifestyle variables

The adolescents answered a food frequency questionnaire (FFQ) on dietary habits, tobacco and alcohol use, and oral hygiene habits at their visit to the dental clinic. For the adults, diet information was obtained from the FFQ included in the questionnaire at the health screening [26]. In both cohorts, the FFQ provided 9 intake frequency options. The FFQ used in the replication cohort has been validated [26], and the FFQ used for the adolescents were identical to that used in the replication cohort with addition of questions on vegetarian foods and non-sugar sweetened products. Quality filtering of the FFQ data were as described [9]. In brief, participants were excluded from analysis if FFQ answers were incomplete, if the reported Food Intake Level (FIL) was extreme with an unrealistic ratio between estimated energy intake and need. Tobacco use [smoking or Swedish snuff (snus)] was classified as current, former or never use, tooth brushing (adolescents only) as ≥1 a day or less, education (adults only) as having an academic education or not, BMI was calculated as weight (kg)/height (m)^2^.

### Cultivation and PCR detection of *S*. *mutans* and *S*. *sobrinus* in the study cohort

Viable mutans streptococci (*S*. *mutans* and *S*. *sobrinus*) and lactobacilli were assessed in fresh saliva from the adolescents by cultivation on mitis salivarius sucrose agar supplemented with 0.2 U of bacitracin (MSB) and Rogosa agar (Merck, Darmstadt, Germany), respectively. The plates were incubated at 37°C in 5% CO_2_ for 48 h. Presence of *S*. *mutans* and *S*. *sobrinus* in tooth biofilm and saliva was evaluated by PCR using the KAPA2G Robust HotStart PCR Ready Mix (2´) kit (Kapa Biosystems, Boston, MA, USA) and the primers SmF5 (5′-ACTACACTTTCGGGTGGCTTGG-3′ and SmR4 (5′-CAGTATAAGCGCCAGTTTCATC-3′) primers for *S*. *mutans* and SsF3 (5′-GATAACTACCTGACAGCTGACT-3) and SsR1 (5′AAGCTGCCTTAAGGTAATCACT-3′) primers for *S*. *sobrinus* as described previously [27].

### DNA extraction and Illumina MISeq sequencing in the study cohort

DNA was extracted from 154 saliva and 139 tooth biofilm samples from the adolescents. The procedure for DNA extraction and sequencing, and overall findings from sequencing have been presented previously [22]. Briefly, genomic DNA was extracted from tooth biofilm and saliva samples using the GenElute™ Bacterial Genomic DNA Kit (Sigma-Aldrich, St. Louis, MO, USA), and samples with a 260/280 ratio of the extracted DNA of ≥1.8 was used, and adjusted to 20 ng/μl by lyophilization of a fixed amount of DNA and resolving in a defined volume. Multiplex 16S rDNA amplicon sequencing was performed using the Illumina MiSeq platform (http://www.illumina.com) at the Forsyth Research Institute (Cambridge, MA, USA) using the HOMI*NGS* protocol [28]. The V3-V4 hypervariable regions of the 16S rRNA gene were PCR amplified using the 341F forward primer AATGATACGGCGACCACCGAGATCTACACTATGGTAATTGT***CCTACGGGAGGCAGCAG*** and the 806R reverse primer CAAGCAGAAGACGGCATACGAGATNNNNNNNNNNNNAGTCAGTCAGCC***GGACTACHVGGGTWTCTAAT***. The sequences in bold-italic represent the primers and the 12 N stretch designate individual barcode sequences (http://homings.forsyth.org/protocol.html). Ultrapure water served as the negative control and three mock communities as positive controls as described previously [22].

Pair-end reads were combined with an overlap of 70 bp and mismatch ratio of 0.25, and barcodes, primers, and ambiguous and chimeric sequences were removed. Taxa were identified by in silico recognition of 17–40 bp species- and genus-level 16S rRNA-based oligonucleotide “probes” using ProbeSeq (a custom designed BLAST program); 638 species- and or 129 genus-level probes for closely related species [29]. Taxa targeted by the species and genus probes (gp) are described in S1 Table and S2 Table and at http://homings.forsyth.org/index2.html.

As described previously [22], 9,744,159 reads with a mean read length of 426 bp were retained for 152 saliva samples (2 samples failed), and 4,829,569 reads (with an average read length of 424 bp) for the 139 tooth biofilm samples. The average (min-max) number of included sequences per sample was 64,106 (21,951–157,126) for saliva (n = 152 samples) and 34,745 (8,237–109,373) for tooth biofilm samples (n = 139). Taxa were found in 85 genera (9 phyla) in saliva and 81 genera (10 phyla) in tooth biofilm. In total, 311 and 323 species were identified in saliva and tooth biofilm, respectively. All sequences can be found at 10.6084/m9.figshare.5794989.

### Caries scoring

In the adolescents, dental caries was scored from visual and radiographic examinations [22]. The sum of manifest, cavitated lesions and initial, non-cavitated caries in the enamel (D_1_) and filled (F) tooth surfaces was calculated (D_1_FS scores). No participants had lost any tooth due to caries, i.e. the D_1_FS scores corresponded to a DMFS score with M = 0.

For the adults in the replication cohort, information on caries prevalence (DMFS scores, i.e. the sum of decayed (manifest cavitated lesions), missing, and filled tooth surfaces for 32 teeth) was retrieved from electronic dental records and linked to questionnaire data using personal reference numbers, which uniquely identify Swedish citizens.

### Statistical analyses

The adolescents in the study group were ranked into tertile groups and the adults in the replication group into quintile groups based on their self-reported total milk (non-fermented+fermented milk), non-fermented and fermented milk intakes, respectively. Ranking was done within sex subgroups for the adolescents and sex and 10-year age subgroups for the adults.

Continuous, normally distributed variables are presented as means with 95% confidence limits (95% CI) adjusted by general linear modelling (GLM) for potential confounders as specified in the legend or footnotes of the tables. Categorical variables are presented as percentages. Differences between group means were tested with ANOVA in GLM after appropriate covariate standardization; differences in group distributions were tested using Chi^2^ tests. Trends across the strata of milk consumption were assessed using the non-parametric Jonckheere-Terpstra trend test. These analyses were performed using SPSS version 24 (IBM Corporation, Armonk, NY, USA) with p-values <0.01 considered statistically significant.

For the Illumina MiSeq sequences each taxa was scored for detection (yes/no) and relative abundance (percentage of all sequences in the individual). Univariate comparisons of relative taxa abundances were done with non-parametric Mann-Whitney test and p-values were corrected for multiple comparisons by the false discovery rate (FDR). Association between bacterial taxa (in saliva and tooth biofilm) and tertiles of milk consumption was assessed using multivariate partial least squares (PLS) regression (SIMCA P+ version 12.0, Umetrics AB, Umeå, Sweden). The PLS models included the milk tertile allocation as a block of dependent variables and all taxa identified by sequencing as the independent blocks. The results are presented in PLS scatter plots for subject clustering and PLS correlation coefficients from PLS column loading plots for being in the highest versus lowest milk tertile group. Taxa with a statistically significant correlation or a correlation coefficient ≥0.1 are presented. PLS modelling was also done with lifestyle variables as the independent block. The relative importance of each x-variable in PLS scatter loading plots is expressed by variable importance in the projection (VIP) values. VIP-value ≥1.0 are considered influential and ≥1.5 as highly influential. Variables utilized for PLS regression were auto-scaled and logarithmically transformed as needed to improve normality.

### Ethical approval and consent to participate

Both studies have been approved by the Regional Ethical Board, at Umeå university, Sweden with registration number: Dnr 2012-111-31M with an addendum (Dnr 2015-389-32M) for the adolescents, and Dnr 2010-387-31M and 2011-74-32M for the adults. All participants signed an informed consent to participate and that the information could be used for research. The guardians of the adolescents´ approved their participation.

## Results

### Study group characteristics

The 154 participating adolescents were allocated into tertile groups based on their reported total milk, non-fermented and fermented milk intake, respectively. The mean intakes for the tertile groups are shown in Table 1 for total milk, and S3 Table for non-fermented and fermented milk. Other characteristics such as sex, tobacco use, BMI and oral hygiene behaviours were similar in the three tertiles, with no statistically significant difference between the strata of milk consumption Table 1.

**Table 1:** Participant characteristics. Data are for 154 adolescents and 31,571 adults allocated into ranking groups by their intake of total milk, i.e., non-fermented plus fermented milk.

	Ranking groups from total milk intake					
**Adolescents**^1^	**Lowest tertile**		**Middle tertile**		**Highest tertile**	**p-value between groups**
Numbers	51		53		50	
Males, %	43.1		43.4		46.0	0.950
Total milk. servings/day, mean (95% CI)^2^	0.41 (0.34, 0.48)		1.54 (1.36, 1.72)		3.66 (3.36, 3.96)	<0.001
Present smoker, %	4.0		1.9		2.0	0.755
Present snuff user, %	2.0		3.8		2.0	0.802
BMI, kg/m^2^ mean (95% CI)^2^	21.6 (20.6, 22.5)		22.5 (21.5, 23.4)		22.1 (21.2, 23.1)	0.400
Tooth brushing, % ≥1 daily	88.2		79.2.0		84.0	0.461
Caries, D_1_FS, mean (95% CI)^3^	6.2 (4.3, 8.1)		5.3 (3.4, 7.2)		4.5 (2.6, 6.4)	0.460
**Adults**^1^	**Lowest quintile**	**2**^**nd**^ **quintile**	**Middle quintile**	**4**^**th**^ **quintile**	**Highest quintile**	
Number	6,323	6,310	6,317	6,310	6,311	
Males, %	49.0	49.0	49.3	49.0	49.0	0.995
Total milk. servings/day, mean (95% CI)^2^	0.42 (0.41, 0.43)	1.08 (1.07, 1.09)	1.57 (1.56, 1.58)	2.39 (2.38, 2.40)	3.67 (3.66, 3.69)	<0.001
Present smoker, %	16.9	14.5	612.1	15.2	16.0	<0.001
Present snuff user, %	19.6	18.9	17.3	18.5	18.3	0.015
BMI, kg/m^2^; mean (95% CI)^2^	26.5 (26.4, 26.6)	26.4 (26.3, 26.5)	26.2 (26.1, 26.3)	26.4 (26.3, 26.5)	26.5 (26.4, 26.6)	0.003
Education, % university	23.2	23.7	28.0	25.2	24.7	<0.001
Caries, DFS, mean (95% CI)^4^	36.0 (35.5,36.4)	35.8 (35.4, 36.3)	35.9 (35.5, 36.4)	35.8 (35.4, 36.3)	35.7 (35.3, 36.1)	0.986
Caries, DMFS, mean (95% CI)^4^	62.9 (62.4, 63.5)	62.1 (61.5. 62.6)	62.5 (61.9, 63.0)	63.0 (62.5, 63.6)	63.8 (63.3, 64.3)	<0.001
Missing teeth mean (95% CI)^4^	5.5 (5.4, 5.6)	5.3 (5.2, 5.4)	5.4 (5.3, 5.5)	5.5 (5.4, 5.6)	5.7 (5.6, 5.8)	<0.001

1) Adolescents were ranked into tertile groups based on their reported total milk intake in sex strata. Adults were ranked into quintiles groups based on their reported total milk intake in sex and 10-year age-group strata.

2) Means and 95% CI limits adjusted for sex in the adolescents and for sex, and age in adults.

3) The caries data are for 28 teeth in the adolescents and means and 95% CI limits are adjusted for sex and tooth brushing. The means after additional adjustment for snacking frequency were 6.1, 5.1, and 4.7 (p = 0.603). There was no evidence for interaction between snacking frequency and milk intake.

4) The caries data are for 32 teeth in the adults, and means and 95% CI limits are adjusted for sex, age (continuous), education, smoking and examination year. Cases which had a missing value for one or more of the covariates were excluded. The means after additional adjustment for snacking frequency were 62.9, 62.0, 62.3, 62.7 and 63.2 (p = 0.020). There was no evidence for interaction between snacking frequency or sugar intake and milk intake.

### Milk intake and tooth biofilm microbiome

PLS multivariate regression was used to identify if milk intake was associated with participant clustering based oral bacteria profiles. As a first step, PLS regression of bacterial taxa identified in saliva and tooth biofilm, respectively, was performed with all three tertiles of total milk intake as dependent variables (S1 Fig and S2 Fig). These models indicated that milk consumption was associated with different taxa profiles in saliva and tooth biofilms, respectively, with pronounced differences between the highest and lowest tertiles.

**Fig 1:**
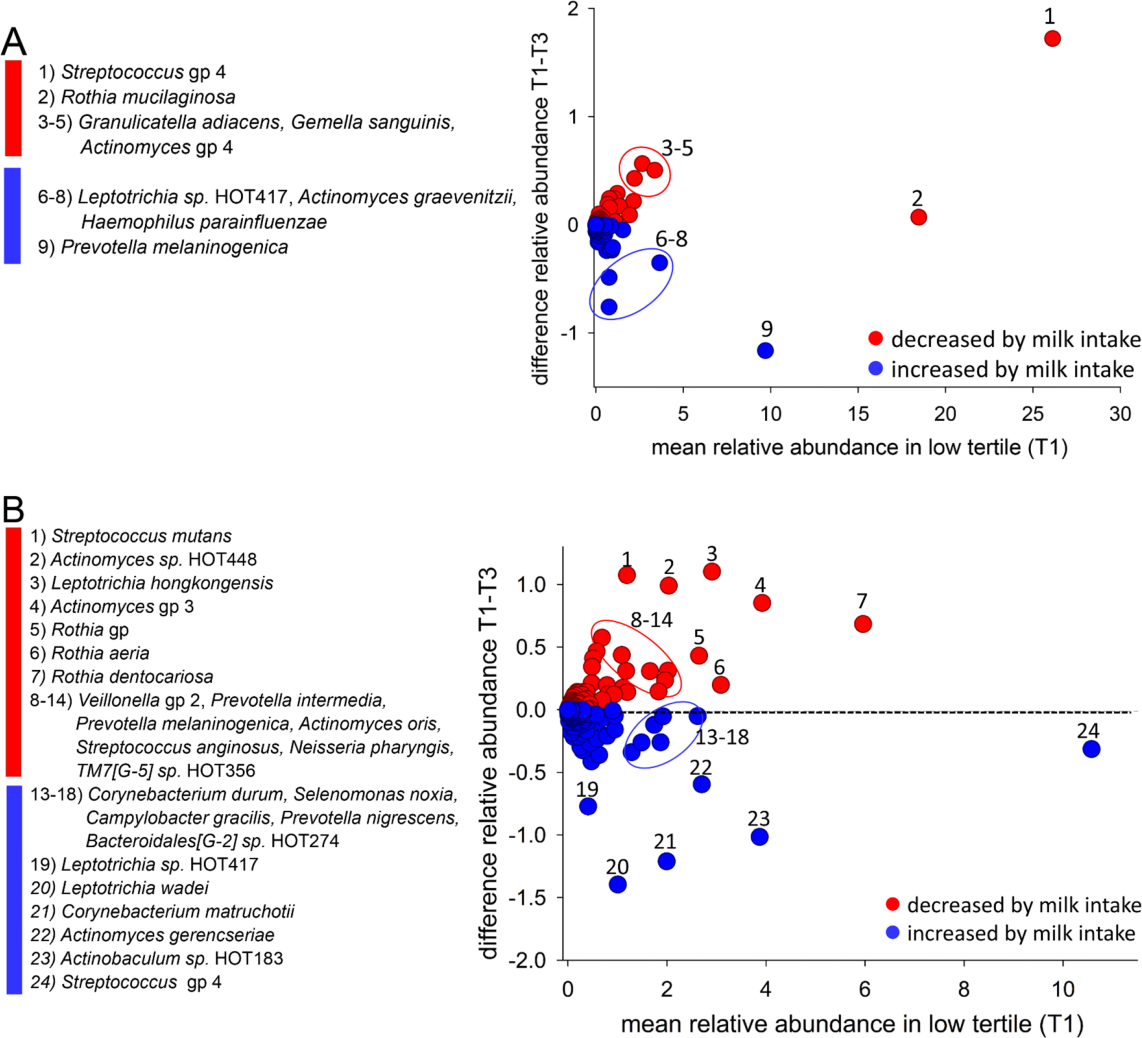
Bland-Altman plots. The plots show mean taxa abundances for the lowest milk intake group on the x-axis and the mean abundance difference between the lowest and highest tertile groups on the y-axis for A) saliva and B) tooth biofilm.

Taxa with the highest relative abundance in the low milk tertile and the difference against the high tertile are illustrated in Bland-Altman plots for saliva (Fig 1A) and tooth biofilm (Fig 1B). *Streptococcus* gp 4 recognised taxa dominated in both sample types, followed by *Rothia mucilaginosa and Prevotella melaninogenica* in saliva and *Rothia dentocariosa* in tooth biofilm. Among the indicated taxa, only *S*. *mutans* in tooth biofilm differed significantly after FDR correction. i.e., p<0.008, Less abundant taxa that differed significantly between the low and high milk tertiles in the univariate comparisons were *Actinomyces sp*. HOT448 for saliva, and *Butyrivibrio sp*. HOT080, *Peptostreptococcaceae*[XI][G-7] *sp*. HOT081, *Prevotella sp*. HOT472 and *TM7*[G-1] *sp*. HOT348 for tooth biofilm.

**Fig 2:**
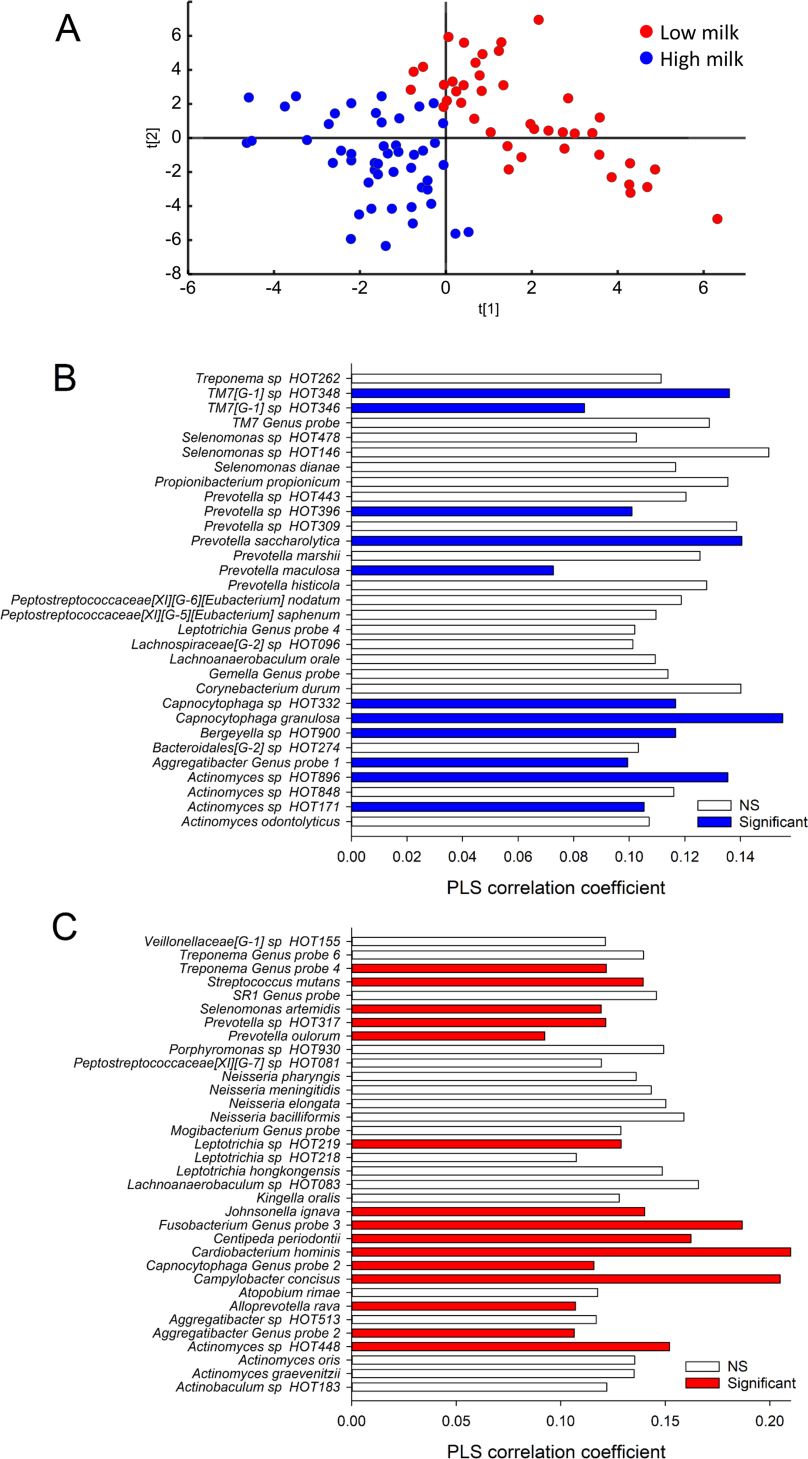
Participant clustering and saliva taxa associated with milk intake. (A) PLS separation of participants based on saliva microbiota characterization by Illumina MiSeq sequencing displayed in a PLS score scatter plot. The scores t1 and t2 on the x- and y-axes are the new created variables summarizing the *x* variables. Red dots indicate adolescents in the lowest tertile of total milk intake, and blue dots those in the highest tertile. The two lower bar graphs list taxa with statistically significant correlation or a PLS correlation coefficient ≥0.1 for (B) being in the highest tertile of total milk intake and (C) in the lowest tertile. Colored bars are for taxa with statistically significant PLS correlation coefficients, i.e. the 95% CI does not include zero.

**Fig 3:**
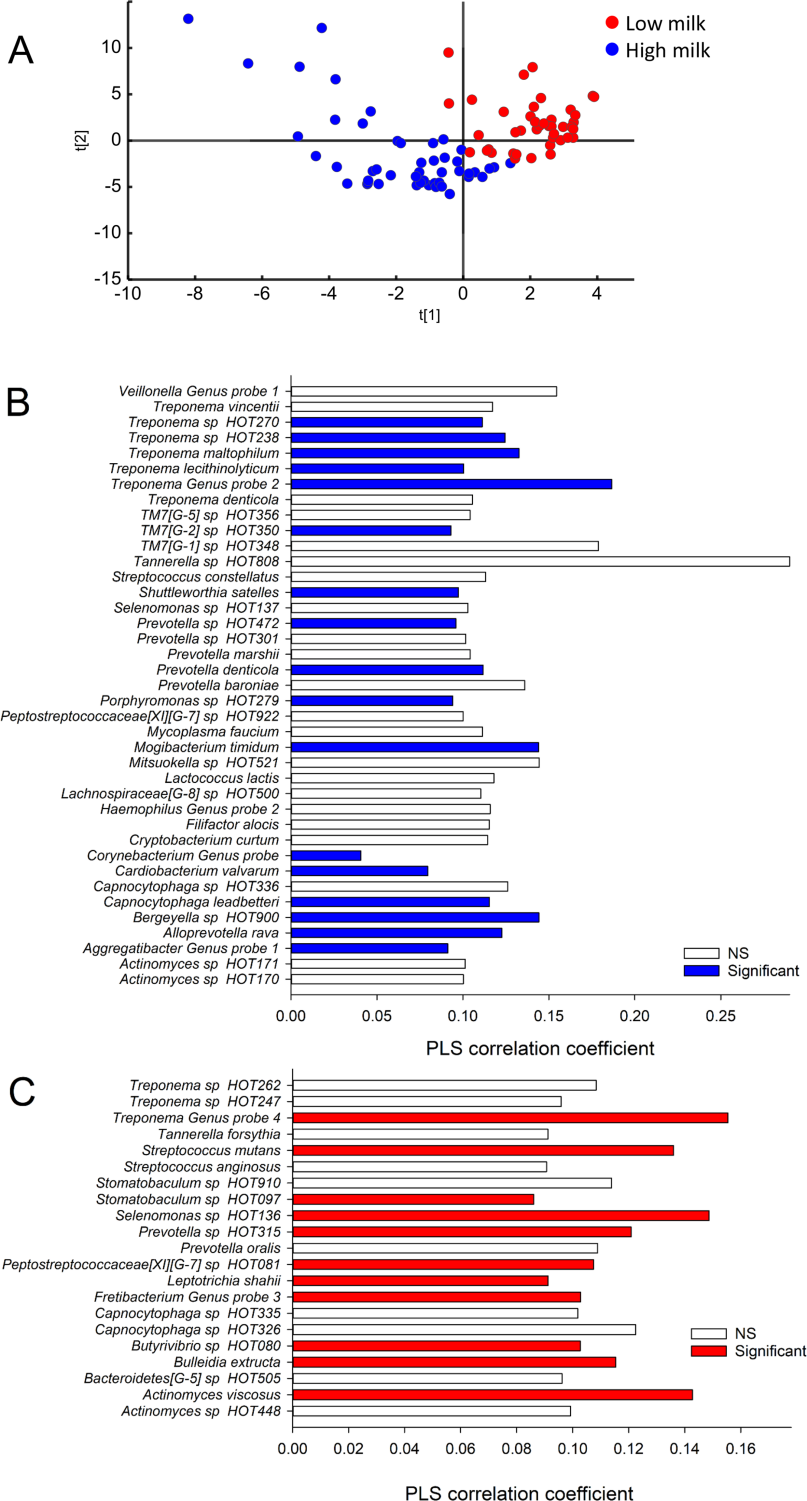
Participant clustering and tooth biofilm taxa associated with milk intake. (A) PLS separation of participants based on tooth biofilm microbiota characterization by Illumina MiSeq sequencing displayed in a PLS score scatter plot. The scores t1 and t2 on the x- and y-axes are the new created variables summarizing the *x* variables. Red dots indicate adolescents in the lowest tertile of total milk intake, and blue dots those in the highest tertile. The two lower bar graphs list taxa with statistically significant correlation or a PLS correlation coefficient ≥0.1 for (B) being in the highest tertile of total milk intake and (C) in the lowest tertile. Colored bars are for taxa with statistically significant PLS correlation coefficients, i.e. the 95% CI does not include zero.

A PLS model restricted to adolescents in the highest and lowest tertiles, performed to search for hidden structures among taxa, separated the adolescents with the highest milk intake from those with the lowest intake by saliva (Fig 2A) and tooth biofilm (Fig 3A) taxa.

For saliva (Fig 2B and 2C), PLS revealed high and low intake of milk as associated with different profiles of species in the genera *Actinomyces*, *Aggregatibacter*, *Capnocytophaga*, *Lachnoanaerobaculum*, *Leptotrichia*, *Prevotella*, *Selenomonas* and *Treponema*. Low intake was specifically associated with more prevalent detection of single species in *Alloprevotella*, *Campylobacter*, *Cardiobacterium*, *Centipedia*, *Fusobacterium*, *Johnsonella*, *Neisseria* and *Streptococcus* (*S*. *mutans)*, compared to species in the candidate division *TM7* [G-1] and *Bergeyella* in high intake.

For tooth biofilm (Fig 3B and 3C) high and low intake of milk was associated with different profiles of species in the genera *Actinomyces*, *Capnocytophaga*, *Peptostreptococcaceae* [XI] [G-7], *Prevotella*, *Selenomonas*, *Streptococcus*, *Tannerella* and *Treponema*. *S*. *mutans* correlated significantly with being in the lowest milk tertile also in tooth biofilms. Further, low intake was specifically associated with more prevalent detection of single species in the genera *Bulleida*, *Butyrivibrio*, *Fretibacterium*, and *Stomatobaculum* compared to species in *Bergeyella*, *Cardiobacterium*, *Corynebacterium*, *Mogibacterium*, *Porphyromonas*, *Shuttleworthia*, and candidate division *TM7* [G-1].

**Fig 4:**
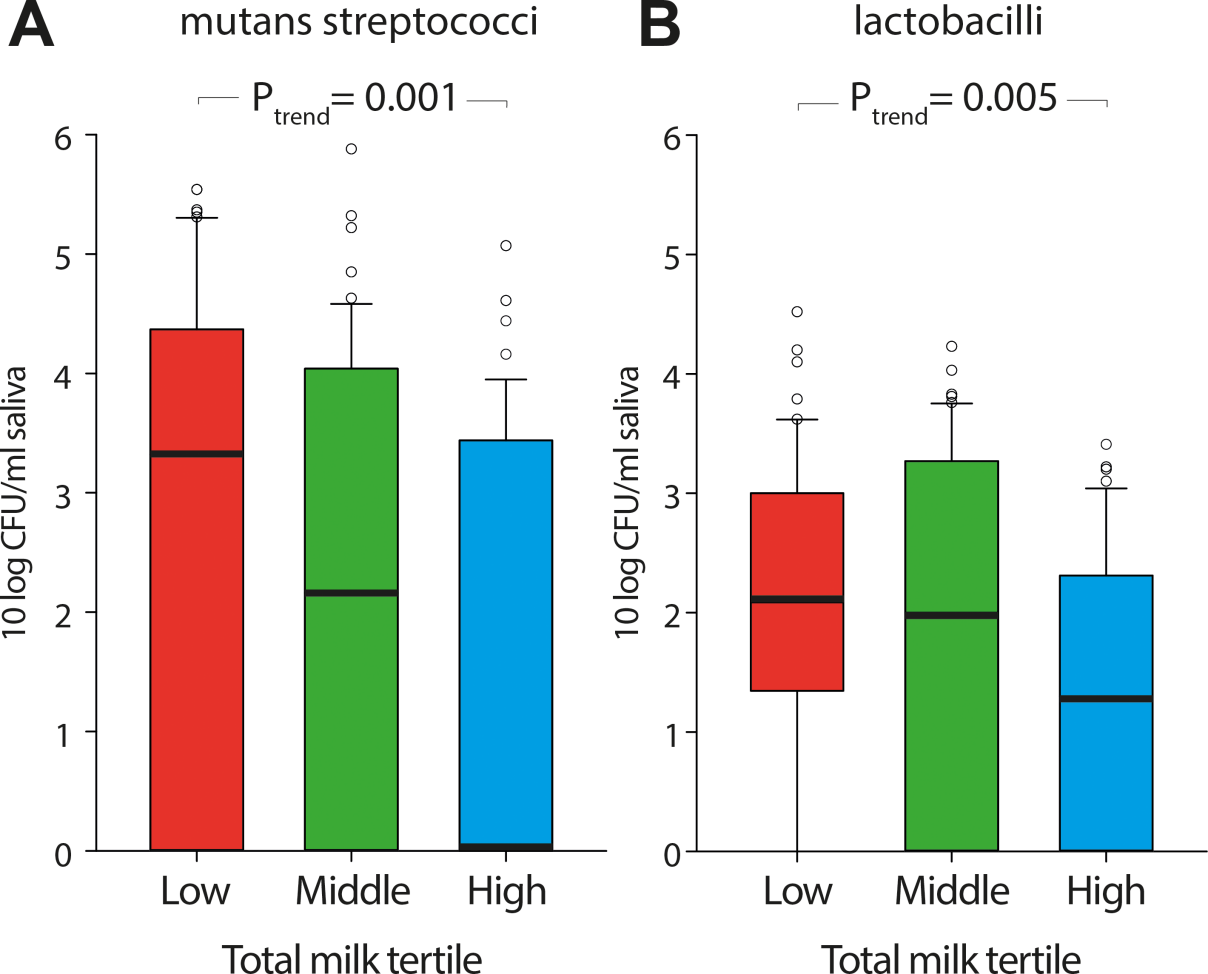
Box plot from mutans streptococci and lactobacilli culture. The plot illustrates colony forming units (CFU) per ml of whole chewing-stimulated saliva of (A) mutans streptococci, and (B) lactobacilli. Data are from culturing on MSB and Rogosa agar, respectively and are expressed as ^10^log counts.

Univariate follow-up revealed that PCR analyses detected *S*. *mutans* more frequently in both saliva and tooth biofilm from adolescents with low milk intake than adolescents with higher milk intake (Table 2). PCR detected *S*. *mutans* in 20% of the tooth biofilm samples from the adolescents with the highest total milk intake compared to 45% in the middle tertile and 51% of those with the lowest intake (p_trend_ = 0.002; Table 2). In agreement with this, increasing milk intake was associated with decreasing trends of viable mutans streptococci in saliva (p_trend_ = 0.001; Fig 4A), and decreasing relative abundance of *S*. *mutans* by Illumina sequencing of both saliva and tooth biofilm samples (p_trend_ = 0.01, p_trend_ = 0.002, respectively) (Table 2). The trends were similar for non-fermented and fermented milk (S3 Table). Further, the numbers of viable lactobacilli decreased with increasing total milk intake (Fig 4B).

**Table 2:** Detection of mutans streptococci by cultivation and DNA based methods in adolescents classified into tertiles based on their reported intake of total milk, i.e., non-fermented milk plus fermented milk.

ADOLESCENTS	Low tertile	Middle tertile	High tertile	p-value trend
**Numbers**	51	53	50	−
**Cultivation for viable bacteria, CFU/ml saliva**				
mutans streptococci, median (interquartile range)	2,080 (23,450)	145 (11,250)	0 (2,760)	0.001
***S*. *mutans* in tooth biofilm by DNA**				
positive by PCR, %	51.0	43.4	20.4	0.002
relative abundance by sequencing, mean (95% CI)^1^	1.19 (0.00, 2.48)	0.84 (0.01, 1,69)	0.12 (0.00, 0.23)	0.002
***S*. *mutans* in saliva by DNA**				
positive by PCR, %	72.5	58.5	53.1	0.010
relative abundance by sequencing, mean (95% CI)^1^	0.09 (0.05, 0.13)	0.06 (0.02, 0.09)	0.07 (0.02, 0.11)	0.011
***S*. *sobrinus* in tooth biofilm by DNA**				
positive by PCR, %	2.0	1.9	2.0	0.978
***S*. *sobrinus* in saliva by DNA**				
positive by PCR, %	2.0	3.8	2.0	0.974

1) Means and 95% CI limits adjusted for sex.

### Milk intake and caries prevalence in 154 adolescents

The finding that the caries related *S*. *mutans* was associated with the level of milk intake urged us to evaluate caries prevalence by milk intake. The sex and tooth brushing adjusted mean caries prevalence (D_1_FS) was numerically lower in the adolescents with the highest milk intake but the difference was not statistically significant (p = 0.460; Table 1). Additional adjustment for BMI did not alter the difference and the result was robust in sensitivity analyses by sex, and *S*. *mutans* infection.

### Sweets snack intake by milk intake in 154 adolescents

**Fig 5:**
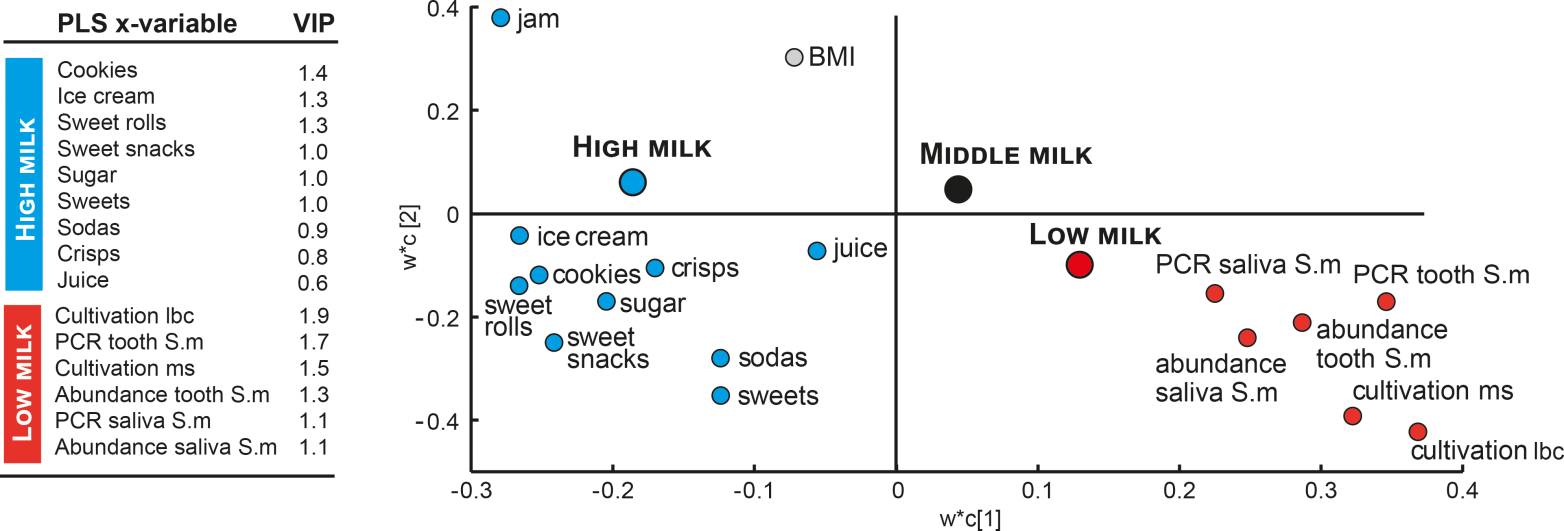
PLS loading score plot. The plot illustrates the association of self-reported sweet and non-sweet snack intake from the questionnaire, BMI, and makers of *S*. *mutans* and lactobacilli in tooth and saliva samples in a PLS model where tertile group allocation from total milk intake were the dependent variables. Variables to the left (blue) were associated with being in the highest tertile and those to the right (red) with being in the lowest tertile. The relative importance of each x-variable in the projection is expressed by a variable importance in the projection (VIP). VIP-value ≥1.0 are considered influential and ≥1.5 as highly influential. VIP-values are listed to the right.

Intake of sweet products was compared between the milk tertile groups in order to search for a potential explanation for the similar caries levels among the milk groups in spite of similar tooth brushing frequency and significantly less *S*. *mutans* infection in the highest consumption group. Higher milk intake was paralleled by higher intake of several sweet products, such as frequency of sugar containing snacks, cookies, sweet rolls, and jam/marmalade (Fig 5, Table 3). Adjustment for reported intake frequency of sweet snacking frequency attenuated the difference among D_1_FS scores across strata of milk consumption slightly and the difference remained non-significant with no evidence for interaction between sweet snack frequency and milk intake (Table 1 footnote).

**Table 3:** Reported intake of snack products in 154 adolescents and 31,571 adults. Data are presented as means with 95% confidence limits adjusted for sex and BMI in adolescents and sex, age, BMI, and education in adults.

	**Servings/day in ranked groups^1^**					
**ADOLESCENTS**^1^	**Lowest** **tertile**		**Middle** **tertile**		**Highest tertile**	**p-value** **for trend**
Sweet snacks	1.26 (0.60, 1.93)		1.18 (0.53, 1.82)		2.36 (1.70, 3.03)	0.011
Cookies	0.14 (0.01, 0.28)		0.14 (0.01, 0.28)		0.40 (0.25, 0.55)	0.001
Sweet rolls	0.09 (0.00, 0.21)		0.10 (0.00, 0.22)		0.30 (0.18, 0,43)	0.037
Jam, marmalade	0.08 (0.04, 0.13)		0.10 (0.05, 0.14)		0.16 (0.12, 0.21)	0.050
Sweets	0.38 (0.21, 0.54)		0.27 (0.12, 0.43)		0.48 (0.32, 0.64)	0.883
Sugar, honey	0.07 (0.00, 0.19)		0.10 (0.00, 0.22)		0.26 (0.14, 0.37)	0.127
Ice cream	0.10 (0.00, 0.22)		0.11 (0.00, 0.23)		0.28 (0.15, 0.41)	0.072
Soft drinks (sugar)	0.29 (0.11, 0.47)		0.21 (0.04, 0.38)		0.43 (0.26, 0.61)	0.112
Juice	0.35 (0.20, 0.50)		0.25 (0.11, 0.39)		0.33 (0.18, 0.48)	0.575
Crisps, nuts	0.20 (0.09, 0.32)		0.25 (0.14, 0.36)		0.35 (0.23, 0.46)	0.152
**ADULTS^1^**	**Lowest** **quintile**	**2**^**nd**^ **quintile**	**Middle** **quintile**	**4**^**th**^ **quintile**	**Highest** **quintile**	
Sweet snacks	1.31 (1.28, 1.34)	1.37 (1.34, 1.40)	1.40 (1.37, 1.43)	1.60 (1.57, 1.63)	1.75 (1.72, 1.78)	<0.001
Cookies	0.19 (0.18, 0.20)	0.20 (0.19, 0.21)	0.22 (0.21, 0.23)	0.24 (0.23, 0.25)	0.27 (0.26, 0.30)	<0.001
Sweet rolls	0.23 (0.22, 0.24)	0.25 (0.24, 0.26)	0.29 (0.28, 0.30)	0.31 (0.30, 0.32)	0.35 (0.34, 0.36)	<0.001
Sweets	0.21 (0.20, 0.22)	0.21 (0.20, 0.22)	0.22 (0.21, 0.22)	0.23 (0.22, 0.23)	0.24 (0.23, 0.24)	<0.001
Sugar, honey, jam	0.48 (0.46, 0.50)	0.52 (0.50, 0.55)	0.53 (0.51, 0.55)	0.68 (0.66, 0.70)	0.75 (0.73, 0.7)	<0.001
Ice cream	0.08 (0.07, 0.08)	0.08 (0.08, 0.09)	0.09 (0.09, 0.10)	0.09 (0.09, 0.10)	0.10 (0.10, 0.10)	<0.001
Soft drinks, juice	0.28 (0.27, 0.29)	0.26 (0.25, 0.27)	0.26 (0.24, 0.27)	0.28 (0.27, 0.29)	0.30 (0.29, 0.31)	<0.001
Crisps, nuts	0.08 (0.08, 0.09)	0.08 (0.08, 0.09)	0.09 (0.08, 0.09)	0.08 (0.08, 0.08)	0.09 (0.08, 0.09)	0.036
Sucrose intake, g/day	22.6 (22.2, 22.9)	23.4 (23.1, 23.8)	25.0 (24.6, 25.3)	26.7 (26.4, 27.1)	29.4 (29.0, 29.8)	

1) Adolescents are ranked into tertile groups based on their reported total milk intake in sex strata, and adults are ranked into quintiles groups based on their reported total milk intake in sex and 10 year age group strata.

**Fig 6:**
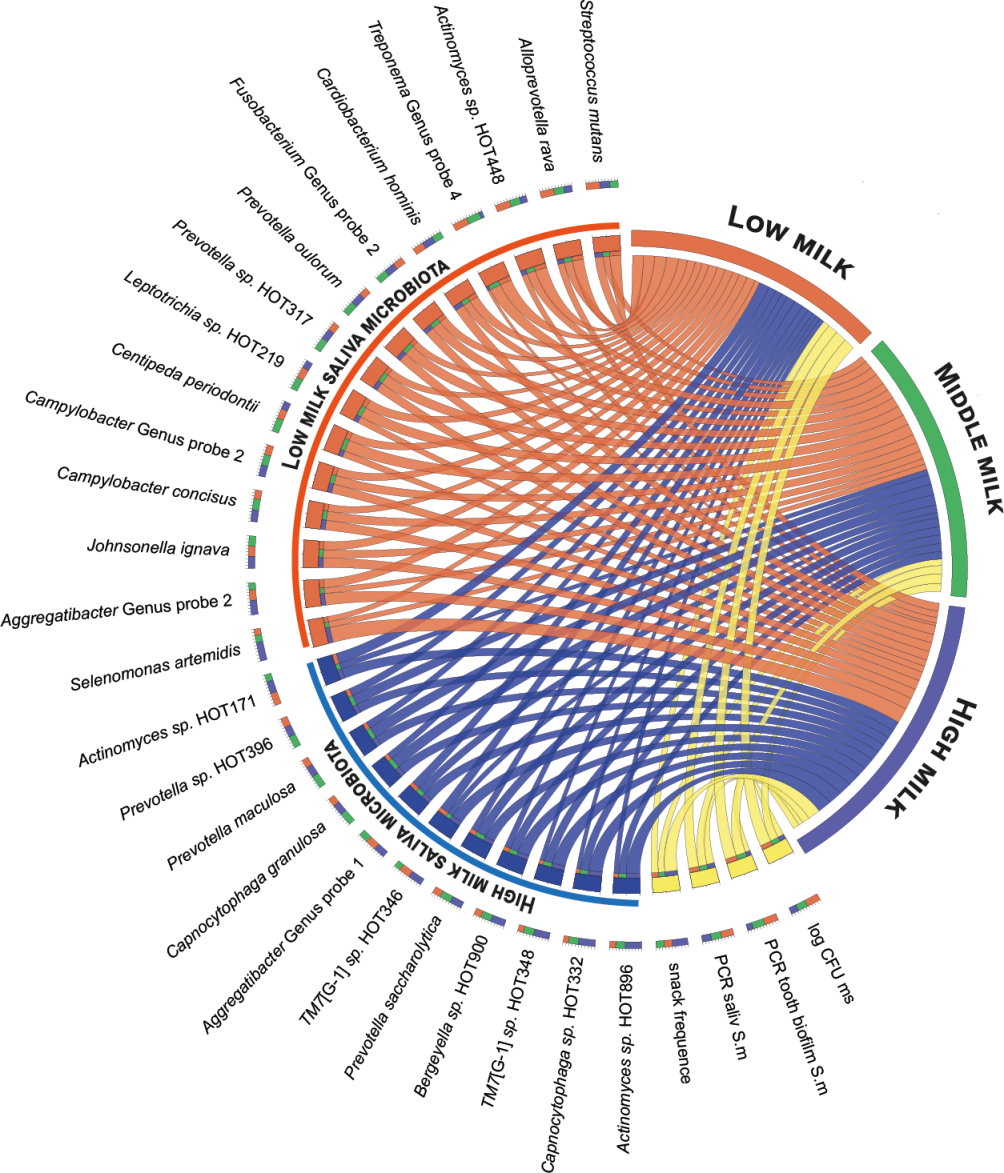
Circos plot for saliva taxa. The plot illustrates the associations between milk intake levels (low, middle, and high tertiles) and saliva taxa differing significantly by milk intake in PLS (cf Figs 1 and 2), frequency of sweet snacks, and PCR detection of *S*. *mutans* (*S*. *m*) and cultivation of mutans streptococci (log CFU ms). Taxa are scaled and listed by relative abundance with those associated with low milk intake highlighted in red and those associated with high intake in blue. The relative abundancies are illustrated by the sizes of each colour segment in the outer circle. The percentages outside the milk group segments refer to the contribution from each of the variables but variable labels are omitted.

**Fig 7:**
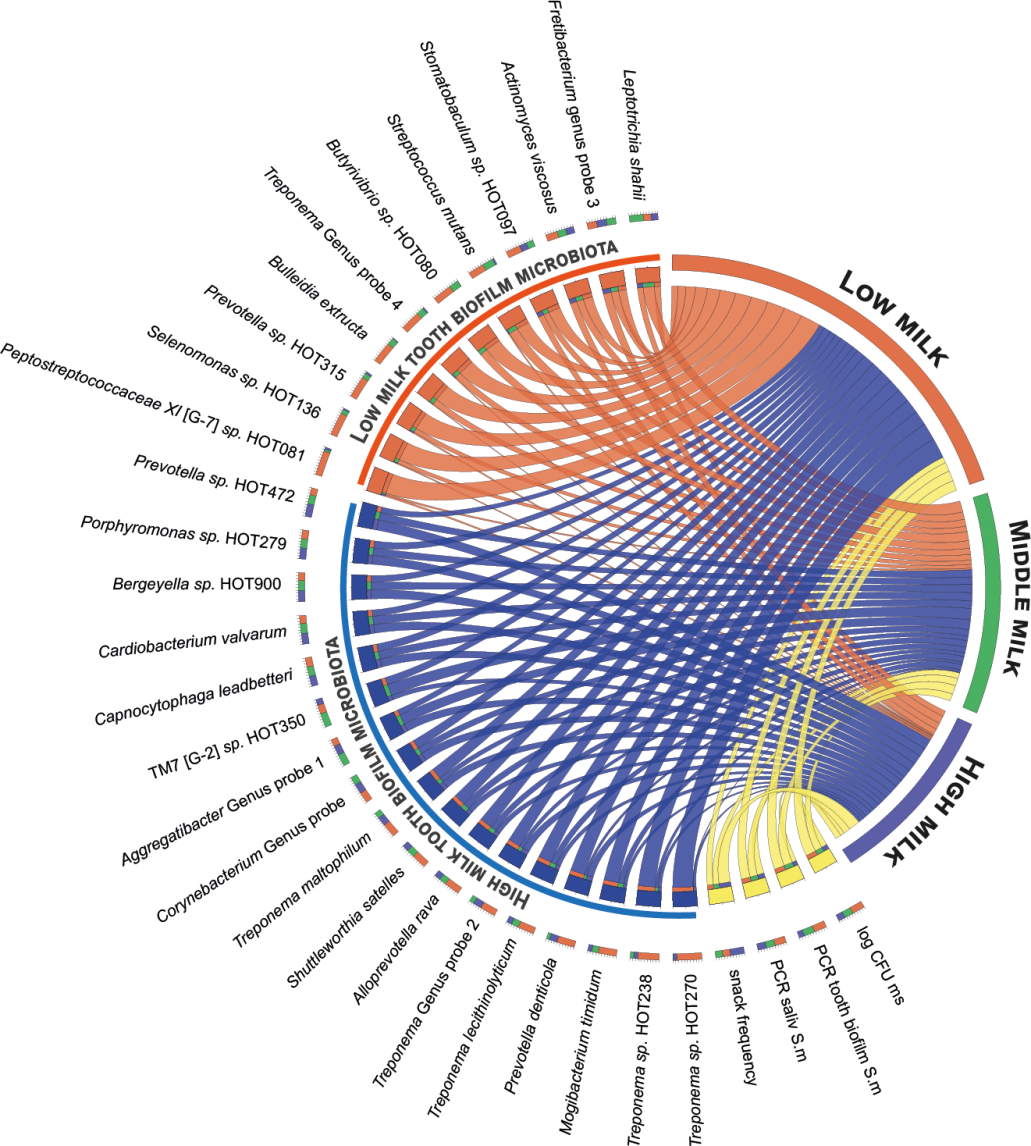
Circos plots for tooth biofilm taxa. The plot illustrates the associations between milk intake levels (low, middle, and high tertiles) and for tooth biofilm taxa differing significantly by milk intake in PLS (cf Figs 1 and 2), frequency of sweet snacks, and PCR detection of *S*. *mutans* (*S*. *m*) and cultivation of mutans streptococci (log CFU ms). Taxa are scaled and listed by relative abundance with those associated with low milk intake highlighted in red and those associated with high intake in blue. The relative abundancies are illustrated by the sizes of each colour segment in the outer circle. The percentages outside the milk group segments refer to the contribution from each of the variables but variable labels are omitted.

The interaction pattern between milk intake level, total snacking frequency and microbial taxa that differed significantly in PLS are illustrated in Circos plots (http://circos.ca/) for saliva and tooth biofilms, respectively (Figs 6 and 7).

### Characteristics of 31,571 adults in the replication cohort

To evaluate a) the lack of association between milk intake and dental caries and b) the positive association between intake of milk and sweet products we searched for replication in a larger cohort of adults with information on dental caries and diet intake available. The adults were ranked into quintile groups based on their reported total milk intake while accounting for sex and age (Table 1). Similarly to the adolescents, there was a large difference in milk consumption between these groups; the quintile with the lowest intake of total milk consumed 0.4 servings per day and the quintile group with the highest intake 3.7 servings per day (Table 1). The difference of other characteristics, such as smoking status, BMI and educational level were statistically significant (p<0.01) across the quintile groups reflecting the large size of these groups rather than biological relevance.

### Sweets snack intake by milk intake in 31,571 adults

The trend of increasing sweet product intake paralleling increasing milk consumption was replicated in the independent adult cohort. Intake frequencies of sugar containing snacks, cookies, sweet rolls, sugar/honey/jam, ice cream and as well as total sugar intake (g/day) increased consecutively with increasing milk quintiles (Table 3).

### Caries prevalence by milk intake in 31,571 adults

In the adults, the DMFS scores (adjusted for sex, age, education, smoking, and examination year) tended to increase by increasing milk intake (p_between groups_<0.001), but with a non-significant trend (p_trend_ = 0.082). The difference in DMFS scores between milk consumption were minimally affected by adjustment for BMI (p<0.001), and sugar intake adjusted for energy (p<0.001) but was somewhat attenuated by adjustment for intake frequency of sweet snacks (p = 0.020, footnote Table 1). The number of missing teeth (out of 32 teeth) was slightly higher in the group with the highest milk intake (Table 1).

## Discussion

Dietary bovine milk consumption has been discussed in relation to health with disparate opinions on health promoting or detrimental effects [5, 30, 31]. The primary aim of the present study was to evaluate the association between bovine milk intake and oral microbiota profile, with the main finding that participants with low milk consumption (less than one serving every second to third day) had distinctly different microbiome profiles in saliva and tooth biofilms compared to those that consumed milk more frequently. For example, species in the *Neisseria* genus were more prevalent in saliva from low milk consumers and the caries associated *S*. *mutans* more prevalent in both saliva and tooth biofilm in the same group. Notably, the variation in milk intake had no penetrance on dental caries in the present study group, which may reflect the complex nature of the disease with *S*. *mutans* being one of several recognized risk factors including exposure to fermentable sugars.

Besides studies on the effects of probiotics in non-fermented/fermented milk [32] we are not aware of any in vivo study in man that describes bovine milk modulating the GI microbiome. The oral cavity is the first part of the GI canal and though there are significant differences between different parts with niche specific microbiomes [33], there are also similarities, such as exposure to components from the diet and the innate immune system and flushing by host secretions [34, 35]. Given that the mouth may, at least partly, serve as a model for the GI tract, it is hypothesized that bovine milk modulates the gut microbiota in line with what is shown for the mouth in the present study and for the mouth and gut for human milk [15, 36]. Such modulations may affect local metabolic events, epithelial barrier leakage and trigger inflammatory responses with further effects on health and disease [11, 37].

Similar to other parts of the GI tract, the mouth is characterized by niche specific bacterial societies determined by the availability of host receptors for bacteria attachment, nutrients, pH and oxygen conditions and competition between bacterial species [35]. Taxa identified in tooth biofilms represent bacteria that are selected by the ability to attach to saliva coated tooth surfaces (acquired pellicle), whereas saliva mirrors bacteria that colonize epithelial surfaces in the mouth as well as teeth. These differences contribute to the taxa profiles for the two sample types studied here, with some species prevalent enough to reach detection level in both sample types, such as *S*. *mutans*, and others in one type only.

The present study found an inverse association between milk intake and *S*. *mutans* colonization, which is in line with previous publications demonstrating *S*. *mutans* adhesion and metabolism blocking effects of bovine milk proteins and peptides [19–22, 38]. In those studies, secretory-IgA, β-casein and lactoferrin (with mapped binding epitopes in the C-terminal section of β-casein and central section of lactoferrin) were responsible for blocking the attachment of *S*. *mutans* cells to saliva coated hydroxyapatite beads (a model for tooth enamel in the mouth) [20]. Milk is a complex fluid with a panel of milk-specific bioactive components, such as the milk fat globular membrane proteins, and others in common for secretions in the mouth and gut, such as secretory-IgA, mucins, lactoferrin, lysozyme and other innate immunity peptides. Proteins and lipids in the fluid phase an on epithelial membranes provide peptide and carbohydrate epitopes for targeted attachment or blocking of free floating bacteria. Other components are bacteriostatic or even bacteriocidal. Thus, there are numerous actions by which bovine milk, similar to human milk, can affect both the oral and gut microbiome, but with the possible exception of *S*. *mutans*, we cannot speculate on what specific components may affect the prevalence of single taxa in high and low milk consumers. One emerging health associated aspect of milk is the A1 versus A2 polymorphism, with A1, with its release of ß-casomorphin opioid peptides, being considered less healthy [39]. In the study area, milk is dominated by A1 but contains both types. It is possible that variation in milk polymorphism contributes to GI microbiome ecology but not colonization of *S*. *mutans* since the binding epitope for *S*. *mutans* in ß-casein was mapped to the C-terminal section distant from the mutation in A1 versus A2 [20]. Thus, there is experimental support for a specific milk effect on *S*. *mutans* colonization, and it may be anticipated that this might be the case for other species too, but we cannot exclude contribution from confounding factors like other dietary components or host genetics.

Though effects of milk on dental caries was not a primary aim of the present study, the inverse association between milk intake and *S*. *mutans* colonization triggered the evaluation of caries by milk intake. Experimental studies have suggested that milk and milk components have cariostatic properties through pH neutralization [40], supersaturation of calcium and phosphate in the tooth interphase [41, 42, 43], and effects on bacteria, but observational studies are inconsistent [44]. Based on these traits combined with our finding of less *S*. *mutans* by increasing milk intake, we expected high milk consumption to be associated with less caries, which was not found. Dental caries is a complex disease with the clinical symptom being tooth tissue demineralization following bacterial secretion of organic acids from sugar fermentation and a drop in local pH [45]. We do not have an immediate explanation for the apparent lack of association between milk intake and caries development, but suggest that any potential protective effect from milk was attenuated by the higher sugar intake. This view was supported by some effect modulation when adjusting for sugar intake. Following that observational studies, such as the present, are influenced by confounding from both socio-economic and host genetic factors, the result should be followed up in a larger study group with greater power and broader monitoring of confounding factors. An alternative would be to use confounding free, milk intake associated genetic variation in lactase persistence in Mendelian randomization to avoid the obstacles in observational studies [46]. Taken together, the present finding of a seemingly lack of effect of milk on caries status illustrates that the role of single risk factors (here, a bacterial species) cannot be easily understood for complex diseases, such as caries, obesity and cardio-metabolic conditions, without also understanding complementary causal or confounding factors [47].

The strengths of the present study include that microbiota sequencing was done in a comparably large group, that the variation in milk intake was wide, that the FFQ monitored the habitual intake during the latest year, and that all participants had been included in the same dental care program from 2–3 years of age. This program includes regular check-ups every 1–1.5 years and preventive measures, including oral hygiene and sugar restriction advices and topical fluoride treatments, based on the clinical status. A further strength was that part of the results was confirmed by independent methods (cultivation and PCR) and other parts were replicated in an independent study group from the same population. Still, there are some weaknesses that should be considered, including recall bias (under-reporting) in the dietary information, that dental data for the adults were available only as an aggregate score across all 32 teeth, and that the mutans streptococci levels were low in the population with approximately 30% being non-colonized and 10% with ≥10^5^ CFU/mL saliva by cultivation. This calls for the study to be repeated in a population with higher colonization variation and then preferentially as a randomized controlled study if a suitable study group can be identified.

It was concluded that milk intake may modulate the saliva and tooth biofilm microbiota. Low intake was associated with higher prevalence of several opportunistic species, including the caries associated *S*. *mutans*. It is hypothesized that milk confers parallel effects in the gut. The study also illustrated that development of complex diseases, such as dental caries, is more elaborate than single bacteria-effects even if they are considered highly influential for the disease, as demonstrated in this study by the lack of association with dental caries due to an assumed confounding by sugar intake.

## Supporting information

S1 Table List of the 638 species-level targets recognizing 538 species in ProbeSeq. Each identified species is accompanied by an Oral Taxon designation (HOT) as defined in the Human Oral Microbiome Database (HOMD) where detailed information is available. The information can also be found at http://homings.forsyth.org/bacterialtaxa.html.
(PDF)

S2 Table List of the 129 genus-level targets for recognition of taxa where identification is limited to a group of several closely related species rather than individual species in the targeted region (*E*. *coli* positions ~341 to 806). The information can also be found at http://homings.forsyth.org/bacterialtaxa.html.
(PDF)

S3 Table Detection of mutans streptococci and lactobacilli in adolescents classified into tertiles based on their reported intake of total milk, non-fermented milk and fermented milk, respectively. (PDF)

S1 Fig PLS loading plot illustrating separation of the tree milk tertiles groups based on bacterial taxa identified by Illumina 16S rDNA sequencing in saliva samples. (TIF)

S2 Fig PLS loading plot illustrating separation of the tree milk tertiles groups based on bacterial taxa identified in tooth biofilm samples by Illumina 16S rDNA sequencing. (TIF)
