# Supplementary material for: Self-reported bovine milk intake is associated with oral microbiota composition
Source: PLoS One. 2018 Mar 21;13(3):e0193504. doi: 10.1371/journal.pone.0193504 (PMC5862454; doi:10.1371/journal.pone.0193504)
Supplement: S1 Table — Each identified species is accompanied by an Oral Taxon designation (HOT) as defined in the Human Oral Microbiome Database (HOMD) where detailed information is available. The information can also be found at http://homings.forsyth.org/bacterialtaxa.html. (PDF) [file pone.0193504.s001.pdf]

SPECIES-LEVEL TARGETS (v2.0)

638 probes recognizing 538 species

* denotes a species that has not been assigned a human oral taxon (HOT) number

Genus species	HOT No.
Abiotrophia defectiva	389
Acinetobacter baumannii	554
Acinetobacter sp oral taxon 408	408
Actinobaculum sp oral taxon 183	183
Actinomyces cardiffensis	850
Actinomyces georgiae	617
Actinomyces gerencseriae	618
Actinomyces graevenitzii	866
Actinomyces israelii	645
Actinomyces johnsonii	849
Actinomyces massiliensis	852
Actinomyces meyeri	671
Actinomyces naeslundii	176
Actinomyces odontolyticus	701
Actinomyces oricola	708
Actinomyces radicidentis	746
Actinomyces sp oral taxon 169	169
Actinomyces sp oral taxon 170	170
Actinomyces sp oral taxon 171	171
Actinomyces sp oral taxon 172	172
Actinomyces sp oral taxon 175	175
Actinomyces sp oral taxon 178	178
Actinomyces sp oral taxon 180	180
Actinomyces sp oral taxon 181	181
Actinomyces sp oral taxon 414	414
Actinomyces sp oral taxon 446	446
Actinomyces sp oral taxon 448	448
Actinomyces sp oral taxon 525	525
Actinomyces sp oral taxon 848	848
Actinomyces sp oral taxon 877	877
Actinomyces sp oral taxon 896	896
Actinomyces sp oral taxon 897	897
Actinomyces timonensis	179
Actinomyces viscosus	688
Aggregatibacter actinomycetemcomitans	531

Aggregatibacter paraphrophilus	720
Aggregatibacter sp oral taxon 458	458
Aggregatibacter sp oral taxon 512	512
Aggregatibacter sp oral taxon 513	513
Agrobacterium tumefaciens	485
Alloiococcus otitis	831
Alloprevotella rava	302
Alloprevotella sp oral taxon 308	308
Alloprevotella sp oral taxon 473	473
Alloprevotella sp oral taxon 474	474
Alloprevotella sp oral taxon 912	912
Alloprevotella sp oral taxon 913	913
Alloprevotella sp oral taxon 914	914
Alloprevotella tannerae	466
Alloscardovia omnicolens	198
Anaerococcus lactolyticus	859
Anaeroglobus geminatus	121
Aquamicrobium lusatiense	660
Arcanobacterium haemolyticum	811
Arsenicicoccus sp oral taxon 190	190
Atopobium minutum	674
Atopobium parvulum	723
Atopobium rimae	750
Atopobium sp oral taxon 199	199
Atopobium sp oral taxon 416	416
Atopobium sp oral taxon 810	810
Atopobium vaginae	814
Bacillus clausii	045
Bacteroidaceae[G-1] sp oral taxon 272	272
Bacteroidales[G-2] sp oral taxon 274	274
Bacteroidales[G-3] sp oral taxon 911	911
Bacteroides heparinolyticus	630
Bacteroides tectus	787
Bacteroides zoogleoformans	465
Bacteroidetes[G-3] sp oral taxon 280	280
Bacteroidetes[G-3] sp oral taxon 281	281
Bacteroidetes[G-3] sp oral taxon 365	365
Bacteroidetes[G-3] sp oral taxon 436	436
Bacteroidetes[G-3] sp oral taxon 503	503
Bacteroidetes[G-3] sp oral taxon 899	899
Bacteroidetes[G-4] sp oral taxon 509	509

Bacteroidetes[G-5] sp oral taxon 505	505
Bacteroidetes[G-5] sp oral taxon 507	507
Bacteroidetes[G-5] sp oral taxon 511	511
Bacteroidetes[G-6] sp oral taxon 516	516
Bdellovibrio sp oral taxon 039	039
Bergeyella sp oral taxon 319	319
Bergeyella sp oral taxon 322	322
Bergeyella sp oral taxon 900	900
Bergeyella sp oral taxon 907	907
Bifidobacteriaceae[G-2] sp oral taxon 407	407
Bifidobacterium animalis subsp animalis	895
Bifidobacterium animalis subsp lactis	895
Bifidobacterium breve	889
Bifidobacterium dentium	588
Bifidobacterium longum	862
Bifidobacterium scardovii	891
Brevundimonas diminuta	590
Bulleidia extructa	603
Butyrivibrio sp oral taxon 094	094
Campylobacter concisus	575
Campylobacter curvus	580
Campylobacter gracilis	623
Campylobacter sp oral taxon 044	044
Campylobacter sputorum	776
Campylobacter ureolyticus	842
Capnocytophaga gingivalis	337
Capnocytophaga granulosa	325
Capnocytophaga haemolytica	627
Capnocytophaga leadbetteri	329
Capnocytophaga ochracea	700
Capnocytophaga sp oral taxon 323	323
Capnocytophaga sp oral taxon 324	324
Capnocytophaga sp oral taxon 332	332
Capnocytophaga sp oral taxon 334	334
Capnocytophaga sp oral taxon 335	335
Capnocytophaga sp oral taxon 336	336
Capnocytophaga sp oral taxon 338	338
Capnocytophaga sp oral taxon 380	380
Capnocytophaga sp oral taxon 412	412
Capnocytophaga sp oral taxon 863	863
Capnocytophaga sp oral taxon 864	864

Capnocytophaga sp oral taxon 878	878
Capnocytophaga sp oral taxon 901	901
Capnocytophaga sp oral taxon 902	902
Capnocytophaga sp oral taxon 903	903
Capnocytophaga sputigena	775
Cardiobacterium hominis	633
Cardiobacterium valvarum	540
Catonella sp oral taxon 451	451
Caulobacter sp oral taxon 002	002
Centipeda periodontii	726
Chlamydophila pneumoniae	733
Chloroflexi[G-1] sp oral taxon 439	439
Clostridiales[F-1][G-1] sp oral taxon 093	093
Clostridiales[F-1][G-2] sp oral taxon 402	402
Clostridiales[F-2][G-1] sp oral taxon 075	075
Clostridiales[F-2][G-2] sp oral taxon 085	085
Clostridiales[F-2][G-3] sp oral taxon 366	366
Clostridiales[F-2][G-3] sp oral taxon 381	381
Clostridiales[F-3][G-1] sp oral taxon 876	876
Corynebacterium diphtheriae	591
Corynebacterium durum	595
Corynebacterium matruchotii	666
Corynebacterium mucifaciens	835
Corynebacterium sp oral taxon 184	184
Corynebacterium urealyticum	853
Cryptobacterium curtum	579
Delftia acidovorans	023
Desulfobulbus sp oral taxon 041	041
Desulfomicrobium orale	703
Desulfovibrio fairfieldensis	605
Desulfovibrio sp oral taxon 040	040
Dialister invisus	118
Dialister micraerophilus	843
Dialister pneumosintes	736
Dialister sp oral taxon 119	119
Dialister sp oral taxon 502	502
Dietzia sp oral taxon 368	368
Dolosigranulum pigrum	813
Eggerthella lenta	654
Eggerthia catenaformis	569
Eikenella corrodens	577

Eikenella sp oral taxon 011	011
Enterococcus faecalis	604
Erysipelothrichaceae[G-1] sp oral taxon 904	904
Erysipelothrichaceae[G-1] sp oral taxon 905	905
Erysipelothrix tonsillarum	484
Erythromicrobium ramosum	747
Eubacterium[11][G-1] infirmum	105
Eubacterium[11][G-1] sulci	467
Eubacterium[11][G-3] brachy	557
Eubacterium[11][G-5] saphenum	759
Eubacterium[11][G-6] minutum	673
Eubacterium[11][G-6] nodatum	694
Eubacterium[11][G-7] yurii	377
Eubacterium limosum	655
Filifactor alocis	539
Finegoldia magna	662
Flavobacteriales[G-1] sp oral taxon 318	318
Flavobacteriales[G-1] sp oral taxon 321	321
Flavobacteriales[G-2] sp oral taxon 320	320
Fretibacterium fastidiosum	363
Fretibacterium sp oral taxon 360	360
Fretibacterium sp oral taxon 361	361
Fretibacterium sp oral taxon 362	362
Fusobacterium gonidiaformans	860
Fusobacterium necrophorum	690
Fusobacterium nucleatum subsp animalis	420
Fusobacterium nucleatum subsp nucleatum	698
Fusobacterium nucleatum subsp polymorphum	202
Fusobacterium nucleatum subsp vincentii	200
Fusobacterium periodonticum	201
Fusobacterium sp oral taxon 205	205
Gardnerella vaginalis	829
Gemella bergeri	555
Gemella haemolysans	626
Gemella morbillorum	046
Gemella sanguinis	757
GN02[G-1] sp oral taxon 871	871
GN02[G-1] sp oral taxon 872	872
GN02[G-2] sp oral taxon 873	873
Granulicatella elegans	596
Haemophilus ducreyi	821

Haemophilus parahaemolyticus	*
Haemophilus parainfluenzae	718
Haemophilus sp oral taxon 035	035
Helicobacter pylori	812
Johnsonella ignava	635
Johnsonella sp oral taxon 166	166
Jonquetella anthropi	777
Kingella kingae	646
Kingella oralis	706
Kingella sp oral taxon 459	459
Lachnoanaerobaculum orale	082
Lachnoanaerobaculum saburreum	494
Lachnoanaerobaculum sp oral taxon 083	083
Lachnoanaerobaculum sp oral taxon 089	089
Lachnoanaerobaculum sp oral taxon 496	496
Lachnoanaerobaculum umeaense	107
Lachnospiraceae[G-2] sp oral taxon 088	088
Lachnospiraceae[G-2] sp oral taxon 096	096
Lachnospiraceae[G-3] sp oral taxon 100	100
Lachnospiraceae[G-5] sp oral taxon 080	080
Lachnospiraceae[G-5] sp oral taxon 455	455
Lachnospiraceae[G-6] sp oral taxon 090	090
Lachnospiraceae[G-7] sp oral taxon 086	086
Lachnospiraceae[G-7] sp oral taxon 163	163
Lachnospiraceae[G-8] sp oral taxon 500	500
Lactobacillus brevis	558
Lactobacillus coleohominis	816
Lactobacillus fermentum	608
Lactobacillus iners	838
Lactobacillus jensenii	839
Lactobacillus kisonensis	424
Lactobacillus parafarraginis	418
Lactobacillus reuteri	818
Lactobacillus salivarius	756
Lactobacillus sp oral taxon 052	052
Lactobacillus vaginalis	051
Lactococcus lactis	804
Lautropia mirabilis	022
Leptothrix sp oral taxon 024	024
Leptothrix sp oral taxon 025	025
Leptotrichia goodfellowii	845

Leptotrichia hongkongensis	213
Leptotrichia shahii	214
Leptotrichia sp oral taxon 212	212
Leptotrichia sp oral taxon 215	215
Leptotrichia sp oral taxon 217	217
Leptotrichia sp oral taxon 218	218
Leptotrichia sp oral taxon 219	219
Leptotrichia sp oral taxon 221	221
Leptotrichia sp oral taxon 223	223
Leptotrichia sp oral taxon 392	392
Leptotrichia sp oral taxon 417	417
Leptotrichia sp oral taxon 462	462
Leptotrichia sp oral taxon 463	463
Leptotrichia sp oral taxon 498	498
Leptotrichia sp oral taxon 847	847
Leptotrichia sp oral taxon 879	879
Leptotrichia wadei	222
Leptotrichiaceae[G-1] sp oral taxon 210	210
Leptotrichiaceae[G-1] sp oral taxon 220	220
Lysinibacillus fusiformis	614
Megasphaera micronuciformis	122
Megasphaera sp oral taxon 123	123
Megasphaera sp oral taxon 841	841
Microbacterium sp oral taxon 185	185
Mitsuokella multacida	684
Mitsuokella sp oral taxon 131	131
Mitsuokella sp oral taxon 521	521
Mobiluncus mulieris	830
Mogibacterium diversum	593
Mogibacterium pumilum	742
Mogibacterium timidum	042
Mollicutes[G-1] sp oral taxon 504	504
Mollicutes[G-2] sp oral taxon 906	906
Mycobacterium leprae	823
Mycoplasma buccale	561
Mycoplasma faucium	606
Mycoplasma fermentans	607
Mycoplasma genitalium	616
Mycoplasma hominis	632
Mycoplasma lipophilum	656
Mycoplasma orale	704

Mycoplasma pneumoniae	732
Mycoplasma salivarium	754
Neisseria bacilliformis	013
Neisseria elongata	598
Neisseria flavescens	610
Neisseria gonorrhoeae	621
Neisseria lactamica	649
Neisseria meningitidis	669
Neisseria pharyngis	729
Neisseria sicca	764
Neisseria sp oral taxon 018	018
Neisseria sp oral taxon 020	020
Neisseria sp oral taxon 499	499
Neisseria sp oral taxon 523	523
Neisseria subflava	476
Neisseria weaveri	092
Olsenella profusa	806
Olsenella sp oral taxon 807	807
Olsenella sp oral taxon 809	809
Olsenella uli	038
Oribacterium sinus	457
Oribacterium sp oral taxon 102	102
Oribacterium sp oral taxon 108	108
Ottowia sp oral taxon 894	894
Paenibacillus sp oral taxon 786	786
Parascardovia denticolens	586
Parvimonas micra	111
Parvimonas sp oral taxon 110	110
Peptococcus sp oral taxon 167	167
Peptococcus sp oral taxon 168	168
Peptoniphilus asaccharolyticus	548
Peptoniphilus indolicus	840
Peptoniphilus lacrimalis	648
Peptoniphilus sp oral taxon 375	375
Peptoniphilus sp oral taxon 386	386
Peptoniphilus sp oral taxon 836	836
Peptostreptococcaceae[11][G-1] sp oral taxon 383	383
Peptostreptococcaceae[11][G-2] sp oral taxon 091	091
Peptostreptococcaceae[11][G-3] sp oral taxon 382	382
Peptostreptococcaceae[11][G-3] sp oral taxon 495	495
Peptostreptococcaceae[11][G-4] sp oral taxon 103	103

Peptostreptococcaceae[11][G-4] sp oral taxon 369	369
Peptostreptococcaceae[11][G-5] sp oral taxon 493	493
Peptostreptococcaceae[11][G-7] sp oral taxon 081	081
Peptostreptococcaceae[11][G-7] sp oral taxon 106	106
Peptostreptococcaceae[13][G-1] sp oral taxon 113	113
Peptostreptococcaceae[13][G-2] sp oral taxon 790	790
Peptostreptococcus anaerobius	542
Peptostreptococcus stomatis	112
Porphyromonas asaccharolytica	547
Porphyromonas catoniae	283
Porphyromonas endodontalis	273
Porphyromonas gingivalis	619
Porphyromonas sp oral taxon 275	275
Porphyromonas sp oral taxon 277	277
Porphyromonas sp oral taxon 278	278
Porphyromonas sp oral taxon 279	279
Porphyromonas sp oral taxon 284	284
Porphyromonas sp oral taxon 285	285
Porphyromonas sp oral taxon 395	395
Porphyromonas uenonis	785
Prevotella baroniae	553
Prevotella bivia	556
Prevotella buccae	560
Prevotella buccalis	562
Prevotella dentalis	583
Prevotella denticola	291
Prevotella enoeca	600
Prevotella fusca	782
Prevotella histicola	298
Prevotella intermedia	643
Prevotella loescheii	658
Prevotella maculosa	289
Prevotella marshii	665
Prevotella melaninogenica	469
Prevotella micans	378
Prevotella multiformis	685
Prevotella multisaccharivorax	794
Prevotella nigrescens	693
Prevotella oralis	705
Prevotella oris	311
Prevotella oulorum	288

Prevotella pallens	714
Prevotella pleuritidis	303
Prevotella saccharolytica	781
Prevotella salivae	307
Prevotella scopos	885
Prevotella shahii	795
Prevotella sp oral taxon 292	292
Prevotella sp oral taxon 293	293
Prevotella sp oral taxon 296	296
Prevotella sp oral taxon 300	300
Prevotella sp oral taxon 301	301
Prevotella sp oral taxon 304	304
Prevotella sp oral taxon 305	305
Prevotella sp oral taxon 306	306
Prevotella sp oral taxon 309	309
Prevotella sp oral taxon 310	310
Prevotella sp oral taxon 315	315
Prevotella sp oral taxon 317	317
Prevotella sp oral taxon 376	376
Prevotella sp oral taxon 396	396
Prevotella sp oral taxon 443	443
Prevotella sp oral taxon 472	472
Prevotella sp oral taxon 475	475
Prevotella sp oral taxon 515	515
Prevotella sp oral taxon 526	526
Prevotella sp oral taxon 820	820
Prevotella veroralis	572
Propionibacterium acidifaciens	191
Propionibacterium acnes	530
Propionibacterium avidum	552
Propionibacterium propionicum	739
Propionibacterium sp oral taxon 192	192
Propionibacterium sp oral taxon 193	193
Propionibacterium sp oral taxon 194	194
Propionibacterium sp oral taxon 915	915
Proteus mirabilis	676
Pseudomonas aeruginosa	536
Pseudomonas fluorescens	612
Pseudomonas otitidis	834
Pseudomonas sp oral taxon 032	032
Pseudoramibacter alactolyticus	538

Pyramidobacter piscolens	357
Rhodocyclus sp oral taxon 028	028
Rothia aeria	188
Rothia dentocariosa	587
Rothia mucilaginosa	681
Scardovia inopinata	642
Scardovia wiggsiae	195
Selenomonas artemidis	124
Selenomonas dianae	139
Selenomonas flueggei	125
Selenomonas noxia	130
Selenomonas sp oral taxon 133	133
Selenomonas sp oral taxon 134	134
Selenomonas sp oral taxon 136	136
Selenomonas sp oral taxon 137	137
Selenomonas sp oral taxon 138	138
Selenomonas sp oral taxon 143	143
Selenomonas sp oral taxon 146	146
Selenomonas sp oral taxon 149	149
Selenomonas sp oral taxon 388	388
Selenomonas sp oral taxon 442	442
Selenomonas sp oral taxon 478	478
Selenomonas sp oral taxon 501	501
Selenomonas sputigena	151
Shuttleworthia satelles	095
Simonsiella muelleri	683
Slackia exigua	602
Sneathia amnionii	844
Sneathia sanguinegens	837
Solobacterium moorei	678
SR1[G-1] sp oral taxon 345	345
SR1[G-1] sp oral taxon 874	874
SR1[G-1] sp oral taxon 875	875
Stenotrophomonas maltophilia	663
Stomatobaculum longum	419
Stomatobaculum sp oral taxon 097	097
Stomatobaculum sp oral taxon 373	373
Stomatobaculum sp oral taxon 910	910
Streptococcus agalactiae	537
Streptococcus anginosus	543
Streptococcus constellatus	576

Streptococcus cristatus	578
Streptococcus downei	594
Streptococcus intermedius	644
Streptococcus mutans	686
Streptococcus parasanguinis II	411
Streptococcus pyogenes	745
Streptococcus sanguinis	758
Streptococcus sobrinus	768
Streptococcus sp oral taxon 064	064
Streptococcus sp oral taxon 066	066
Streptococcus sp oral taxon 068	068
Streptococcus sp oral taxon 069	069
Streptococcus sp oral taxon 431	431
Streptococcus sp oral taxon 486	486
Streptococcus sp oral taxon 487	487
Syntrophomonadaceae[8][G-1] sp oral taxon 435	435
Tannerella forsythia	613
Tannerella sp oral taxon 286	286
Tannerella sp oral taxon 808	808
Tannerella sp oral taxon 916	916
TM7[G-1] sp oral taxon 346	346
TM7[G-1] sp oral taxon 347	347
TM7[G-1] sp oral taxon 348	348
TM7[G-1] sp oral taxon 349	349
TM7[G-1] sp oral taxon 352	352
TM7[G-1] sp oral taxon 353	353
TM7[G-1] sp oral taxon 488	488
TM7[G-2] sp oral taxon 350	350
TM7[G-3] sp oral taxon 351	351
TM7[G-4] sp oral taxon 355	355
TM7[G-5] sp oral taxon 356	356
TM7[G-5] sp oral taxon 437	437
Treponema amylovorum	541
Treponema denticola	584
Treponema lecithinolyticum	653
Treponema maltophilum	664
Treponema pallidum	805
Treponema parvum	724
Treponema pectinovorum	725
Treponema putidum	743
Treponema socranskii	769

Treponema sp oral taxon 226	226
Treponema sp oral taxon 227	227
Treponema sp oral taxon 228	228
Treponema sp oral taxon 230	230
Treponema sp oral taxon 232	232
Treponema sp oral taxon 234	234
Treponema sp oral taxon 235	235
Treponema sp oral taxon 236	236
Treponema sp oral taxon 238	238
Treponema sp oral taxon 239	239
Treponema sp oral taxon 242	242
Treponema sp oral taxon 246	246
Treponema sp oral taxon 247	247
Treponema sp oral taxon 249	249
Treponema sp oral taxon 250	250
Treponema sp oral taxon 252	252
Treponema sp oral taxon 253	253
Treponema sp oral taxon 254	254
Treponema sp oral taxon 255	255
Treponema sp oral taxon 256	256
Treponema sp oral taxon 257	257
Treponema sp oral taxon 258	258
Treponema sp oral taxon 260	260
Treponema sp oral taxon 262	262
Treponema sp oral taxon 263	263
Treponema sp oral taxon 264	264
Treponema sp oral taxon 265	265
Treponema sp oral taxon 268	268
Treponema sp oral taxon 269	269
Treponema sp oral taxon 270	270
Treponema sp oral taxon 271	271
Treponema sp oral taxon 490	490
Treponema sp oral taxon 508	508
Treponema sp oral taxon 517	517
Treponema sp oral taxon 518	518
Treponema vincentii	029
Turicella otitidis	832
Variovorax paradoxus	717
Veillonella atypica	524
Veillonella denticariosi	887
Veillonella dispar	160

Veillonella parvula	161
Veillonella rogosae	158
Veillonella sp oral taxon 780	780
Veillonella sp oral taxon 917	917
Veillonellaceae[G-1] sp oral taxon 129	129
Veillonellaceae[G-1] sp oral taxon 135	135
Veillonellaceae[G-1] sp oral taxon 145	145
Veillonellaceae[G-1] sp oral taxon 148	148
Veillonellaceae[G-1] sp oral taxon 155	155
Veillonellaceae[G-1] sp oral taxon 483	483
Veillonellaceae[G-1] sp oral taxon 918	918
