# Supplementary material for: Self-reported bovine milk intake is associated with oral microbiota composition
Source: PLoS One. 2018 Mar 21;13(3):e0193504. doi: 10.1371/journal.pone.0193504 (PMC5862454; doi:10.1371/journal.pone.0193504)
Supplement: S2 Table — The information can also be found at http://homings.forsyth.org/bacterialtaxa.html. (PDF) [file pone.0193504.s002.pdf]

GENUS-LEVEL TARGETS (v2.0)

129 genus-specific probes (not all species are found in the human oral cavity)

Genus Probe	Species recognized
Achromobacter Genus probe	animicus, denitrificans, pulmonis, ruhlandii, xylosoxidans
Acinetobacter Genus probe	baumannii, calcoaceticus, haemolyticus, johnsonii, junii, lwoffii, parvus, radioresistens, tjernbergiae, sp. oral taxon 408
Actinomyces Genus probe 1	dentalis, sp. oral taxon 449
Actinomyces Genus probe 2	oris, sp. oral taxon 175
Actinomyces Genus probe 3	bovis, bowdenii, catuli, denticolens, graevenitzii, howellii, johnsonii, naeslundii, oris, radicidentis, slackii, timonensis, urogenitalis, viscosus, sp. oral taxon 169, 170, 171, 175, 177
Actinomyces Genus probe 4	canis, cardiffensis, funkei, georgiae, hyovaginalis, meyeri, neuii, odontolyticus, suimastitidis, turicensis, vaccimaxillae, sp. oral taxon 172, 178, 180, 877
Aggregatibacter Genus probe 1	aphrophilus, sp. oral taxon 458
Aggregatibacter Genus probe 2	segnis, sp. oral taxon 512
Alloprevotella Genus probe	rava, tannerae, sp. oral taxon 308, 473, 474, 914
Anaerococcus Genus probe 1	prevotii, tetradius
Anaerococcus Genus probe 2	hydrogenalis, lactolyticus, octavius, prevotii, tetradius, vaginalis
Aquamicrobium Genus probe	defluvii, lusatiense
Arcanobacterium Genus probe	haemolyticum, phocae, pluranimalium
Atopobium Genus probe	fossor, minutum, parvulum, rimae, sp. oral taxon 199
Bacteroides Genus probe	cellulosilyticus, coprophilus, denticanoris, heparinolyticus, plebeius, pyogenes, salanitronis, suis, tectus, zoogleoformans
Bacteroidetes[G-3] Genus probe	sp. oral taxon 280, 281, 365, 436, 503
Bacteroidetes[G-5] Genus probe	sp. oral taxon 505, 507, 511
Bartonella Genus probe	australis, bacilliformis, bovis, capreoli, chomelii, clarridgeiae, henselae, koehlerae, quintana, schoenbuchensis, weissi, sp. oral taxon 001
Bifidobacterium Genus probe 1	adolescentis, angulatum, animalis subsp. animalis, animalis subsp. lactis, bifidum, boum, breve, catenulatum, choerinum, dentium, gallicum, longum subsp. infantis, longum subsp. longum, longum subsp. suis, merycicum, pseudocatenulatum, pseudolongum subsp. globosum, pseudolongum subsp. pseudolongum, ruminantium, thermacidophilum, thermophilum
Bifidobacterium Genus probe 2	asteroides, coryneforme, gallinarum, indicum, minimum, pullorum, saeculare, scardovii, simiae, subtile
Bordetella Genus probe	bronchiseptica, holmesii, parapertussis, pertussis
Brevundimonas Genus probe	diminuta, ikaite, intermedia, mediterranea, poindexterae, vancanneytii, Nitrobacteria hamadaniensis, Nitrobacteria iranicum

Burkholderia Genus probe	ambifaria, anthina, arboris, cenocepacia, cepacia, lata, mana, multivorans, pyrrocinia, stabilis, tropica, ubonensis, vietnamiensis
Campylobacter Genus probe 1	rectus, showae
Campylobacter Genus probe 2	coli, concisus, cuniculorum, curvus, fetus, gracilis, helveticus, hominis, hyointestinali, jejuni, lanienae, lari, rectus, showae, sputorum, subantarcticus, upsaliensis, ureolyticus, sp. oral taxon 044
Capnocytophaga Genus probe 1	infantium, sp. oral taxon 326
Capnocytophaga Genus probe 2	infantium, leadbetteri, ochracea, sputigena, sp. oral taxon 323, 326, 332, 334, 335, 336, 412, 864, 878, 901, 902, 903
Capnocytophaga Genus probe 3	gingivalis, granulosa, haemolytica, sp. oral taxon 338, 863
Cardiobacterium Genus probe	hominis, valvarum
Catonella Genus probe	morbi, sp. oral taxon 164, 451
Chlamydophila Genus probe	abortus, pecorum, pneumoniae, psittaci
Corynebacterium Genus probe	amycolatum, aquilae, atypicum, aurimucosum, caspium, confusum, diphtheriae, durum, freneyi, genitalium, massiliensis, mastitidis, matruchotti, minutissimum, pseudogenitalium, pseudotuberculosis, simulans, singulare, spheniscorum, striatum, sundsvallense, thomssenii, urealyticum, variabile, xerosis
Desulfobulbus Genus probe	elongatus, mediterraneus, propionicus, rhabdoformis, sp. oral taxon 041
Dialister Genus probe 1	micraerophilus, pneumosintes, sp. oral taxon 502
Dialister Genus probe 2	invisus, propionicifaciens, sp. oral taxon 119, sp. oral taxon 502
Dietzia Genus probe	cinnamea, kunjamensis, maris, papillomatosis, sp. oral taxon 368
Eggerthella Genus probe	lenta, sinensis
Enterococcus Genus probe 1	durans, faecium
Enterococcus Genus probe 2	italicus, saccharolyticus
Enterococcus Genus probe 3	aquimarinus, avium, canintestini, casseliflavus, cecorum, dispar, durans, faecium, flavescens, gallinarum, hirae, italicus, malodoratus, pseudoavium, raffinosus, saccharolyticus, sulfureus, thailandicus
Erysipelothrichaceae Genus probe	sp. oral taxon 904, 905
Erysipelothrix Genus probe	rhusiopathiae, tonsillarum
Escherichia Genus probe	albertii, coli, fergusonii
Eubacterium Genus probe 1	brachy, infirmum, saphenum, sulci
Eubacterium Genus probe 2	minutum, nodatum
Filifactor Genus probe	alocis, villosus
Fretibacterium Genus probe 1	sp. oral taxon 358, 453
Fretibacterium Genus probe 2	sp. oral taxon 359, 452
Fretibacterium Genus probe 3	fastidiosum, sp. oral taxon 358, 359, 360, 361, 362, 452, 453
Fusobacterium Genus probe 1	naviforme, nucleatum subsp. fusiforme

Fusobacterium Genus probe 2	naviforme, nucleatum subsp. animalis, nucleatum subsp. vincentii, sp. oral taxon 205
Fusobacterium Genus probe 3	canifelinum, nucleatum subsp. animalis, nucleatum subsp. nucleatum, nucleatum subsp. polymorphum, simiae, sp. oral taxon 203, 370
Fusobacterium Genus probe 4	canifelinum, naviforme, necrophorum, nucleatum subsp. animalis, nucleatum subsp. fusiforme, nucleatum subsp. nucleatum, nucleatum subsp. polymorphum, nucleatum subsp. vincentii, periodonticum, simiae, sp. oral taxon 203, sp. oral taxon 205, sp. oral taxon 370
Gemella Genus probe	asaccharolytica, bergeri, cuniculi, haemolysans, morbillorum, palaticanis, sanguinis
Granulicatella Genus probe	adiacens, paradiacens
Haemophilus Genus probe 1	aegyptius, influenzae
Haemophilus Genus probe 2	haemolyticus, sp. oral taxon 036
Haemophilus Genus probe 3	aegyptius, haemolyticus, influenzae, quentini, sp. oral taxon 036, 908
Helicobacter Genus probe	acinonychis, apodemus, aurati, baculiformis, bizzozeronii, canadensis, cetorum, equorum, felis, ganmani, heilmannii, mesocricetorum, muricola, muridarum, mustelae, pullorum, pylori, rappini, salomonis, suis, trogontum, tursiopsae
Johnsonella Genus probe	ignava, sp. oral taxon 166
Kingella Genus probe 1	denitrificans, sp. oral taxon 012
Kingella Genus probe 2	denitrificans, sp. oral taxon 012
Kytococcus Genus probe	aerolatus, sedentarius
Lachnoanaerobaculum Genus probe	orale, saburreum
Lactobacillus Genus probe 1	gasseri, johnsonii
Lactobacillus Genus probe 2	animalis, apodemi, coleohominis, frumenti, murinus, panis, pontis, reuteri, ruminis, secaliphilus, vaginalis, sp. oral taxon 052
Lactobacillus Genus probe 3	antri, buchneri, casei, coryniformis, curvatus, diolivorans, farraginis, hilgardii, kefiri, kisonensis, oris, paracasei, parafarraginis, parakefiri, rapi, reuteri, rhamnosus, sakei, sunkii, vaginalis, zeae
Lactobacillus Genus probe 4	acidophilus, crispatus, helveticus
Lactobacillus Genus probe 5	paraplantarum, pentosus, plantarum
Leptotrichia Genus probe 1	buccalis, sp. oral taxon 225
Leptotrichia Genus probe 2	hofstadii, sp. oral taxon 909
Leptotrichia Genus probe 3	buccalis, hofstadii, wadei, sp. oral taxon 225, 417, 462, 463, 909
Leptotrichia Genus probe 4	hongkongensis, shahii, trevisanii, sp. oral taxon 212, 215, 217, 223, 392, 879
Leptotrichiaceae Genus probe	sp. oral taxon 210, 220
Listeria Genus probe	innocua, ivanovii subsp. ivanovii, ivanovii subsp. londoniensis, marthii, monocytogenes, seeligeri, welshimeri
Lysinibacillus Genus probe	boronitolerans, fusiformis, macroides, massiliensis, odysseyi,

	sphaericus, xylanilyticus
Mitsuokella Genus probe	jalaludinii, multacida
Mobiluncus Genus probe	curtisii subsp. curtisii, curtisii subsp. holmesii, mulieris
Mogibacterium Genus probe	diversum, neglectum, pumilum, timidum, vescum
Moraxella Genus probe 1	catarrhalis, nonliquefaciens
Moraxella Genus probe 2	bovis, bovoculi, caprae, catarrhalis, cuniculi, equi, lacunata, lincolnii, nonliquefaciens, oblonga, ovis
Mycobacterium Genus probe	aemonae, africanum, angelicum, arosiense, asiaticum, avium, bohemicum, caprae, chimaera, colombiense, conspicuum, gastri, gordonae, haemophilum, intracellulare, kansasii, lacus, leprae, lepromatosis, malmoense, marinum, microti, nebraskense, paraffinicum, paratuberculosis, pinnipedii, pseudoshottsii, riyadhense, shottsii, szulgai, tuberculosis, ulcerans
Mycoplasma Genus probe	alkalescens, anseris, arginini, arthritidis, auris, bovoculi, buccale, canadense, cloacale, coccoides, collis, columbinum, conjunctivae, cricetuli, dispar, equirhinis, falconis, faucium, flocculare, gateae, genitalium, gypis, haemocanis, haemofelis, hominis, hyopneumoniae, hyorhinis, hyosynoviae, iguanae, indiense, meleagridis, mobile, neurolyticum, orale, ovipneumoniae, phocicerebrale, phocidae, pneumoniae, salivarium, spumans, subdolum, timone, vulturii, zalophi
Neisseria Genus probe 1	oralis, sp. oral taxon 016
Neisseria Genus probe 2	bacilliformis, cinerea, dentiae, flava, flavescens, gonorrhoeae, iguanae, meningitidis, mucosa, oralis, pharyngis, polysaccharea, shayeganii, sicca, subflava, wadsworthii, weaveri, sp. oral taxon 018, 020
Olsenella Genus probe	profusa, uli, sp. oral taxon 807
Oribacterium Genus probe	sp. oral taxon 078, 372
Paenibacillus Genus probe	abekawaensis, anaericanus, azoreducens, barengoltzii, borealis, brasilensis, chibensis, cineris, cookii, durus, favisporus, fujiensis, ginsengisoli, graminis, illinoisensis, jamilae, lactis, macerans, nematophilus, odorifer, pabuli, peoriae, polymyxa, riograndensis, sanguinis, stellifer, xylanilyticus, sp. oral taxon 048, 786
Parvimonas Genus probe	micra, sp. oral taxon 110, 393
Peptoniphilus Genus probe	asaccharolyticus, duerdenii, gorbachii, harei, indolicus, ivorii, lacrimalis, olsenii, sp. oral taxon 375, 386, 836
Peptostreptococcus Genus probe	anaerobius, russellii, stomatis
Porphyromonas Genus probe 1	circumdentaria, endodontalis, gingivicanis, sp. oral taxon 285, 395
Porphyromonas Genus probe 2	catoniae, sp. oral taxon 277, 278, 284
Porphyromonas Genus probe 3	asaccharolytica, uenonis
Prevotella Genus probe 1	nanceiensis, sp. oral taxon 299
Prevotella Genus probe 2	buccae, buccalis, corporis, dentasini, denticola, loescheii,

	maculosa, multiformis, oralis, oris, saccharolytica, shahii, sp. oral taxon 304, 317, 443, 472, 526, 820
Propionibacterium Genus probe	acidifaciens, acidipropionici, acnes, australiense, avidum, freudenreichii, granulosum, jensenii, microaerophilum, propionicum, thoenii, sp. oral taxon 192, 193
Proteus Genus probe	mirabilis, penneri, vulgaris
Pseudomonas Genus probe	aeruginosa, alcaliphila, anguilliseptica, cannabina, cedrina, cichorii, fluorescens, fragi, fuscovaginae, jessenii, mendocina, monteilii, mosselii, otitidis, panipatensis, plecoglossicida, poae, pseudoalcaligenes, putida, reactans, stutzeri, syringae, viridiflava, sp. oral taxon 032
Rothia Genus probe	aeria, dentocariosa, mucilaginosa
Sanguibacter Genus probe	inulinus, keddieii, suarezii
Scardovia Genus probe	inopinata, wiggsiae
Selenomonas & Centipeda Genus probe	dianae, flueggei, infelix, noxia, periodontii, sp. oral taxon 126, 138, 140, 146, 388, 479, 481, 892
Selenomonas Genus probe 1	noxia, sp. oral taxon 140
Selenomonas Genus probe 2	sputigena, sp. oral taxon 143
Slackia Genus probe	exigua, faecicanis, isoflavoniconvertens
Sphingomonas Genus probe	aquatilis, dokdonensis, echinoides, insulae, melonis, mucosissima, oligophenolica, rhizogenes, yunnanensis, sp. oral taxon 003
SR1 Genus probe	SR1[G-1] sp. oral taxon 345, 874, 875
Staphylococcus Genus probe 1	aureus, gallinarum
Staphylococcus Genus probe 2	pasteuri, warneri
Staphylococcus Genus probe 3	aureus, capitis, caprae, carnosus, cohnii, condimenti, devriesei, epidermidis, haemolyticus, hominis, pasteuri, piscifermentans, saprophyticus, warneri
Streptococcus Genus probe 1	gordonii, sanguinis
Streptococcus Genus probe 2	pneumoniae, pseudopneumoniae
Streptococcus Genus probe 3	salivarius, vestibularis
Streptococcus Genus probe 4	australis, cristatus, infantis, mitis, mitis bv. 2, oligofermentans, oralis, parasanguinis, parasanguinis II, peroris, pneumoniae, pseudopneumoniae, salivarius, sinensis, vestibularis, sp. oral taxon 055, 056, 057, 058, 061, 064, 065, 066, 067, 068, 069, 070, 071, 074, 423, 431, 486
Tannerella Genus probe	forsythia, sp. oral taxon 286, 808, 916
TM7 Genus probe	[G-1] sp. oral taxon 346, 347, 348, 349, 352, 353, 488, [G-2] sp. oral taxon 350, [G-3] sp. oral taxon 351, [G-4] sp. oral taxon 355, [G-5] sp. oral taxon 356, 437
Treponema Genus probe 1	sp. oral taxon 231, 237
Treponema Genus probe 2	medium, vincentii, sp. oral taxon 226, 228, 230, 231, 234, 235, 236, 237, 238
Treponema Genus probe 3	denticola, putidum, sp. oral taxon 246, 247, 249
Treponema Genus probe 4	sp. oral taxon 250, 251, 252, 253, 254, 255, 256, 508, 517, 518

Treponema Genus probe 5	azotonutricium, caldarium, lecithinolyticum, maltophilum, primitia, zuelzerae, sp. oral taxon 258, 260, 490
Treponema Genus probe 6	socranskii subsp. buccale, socranskii subsp. pedis, socranskii subsp. socranskii, sp. oral taxon 268, 269
Veillonella Genus probe 1	atypica, dispar
Veillonella Genus probe 2	atypica, caviae, criceti, dentocariosi, dispar, montpellierensis, parvula, ratti, rodentium, rogosae, sp. oral taxon 780, 917
Veillonellaceae Genus probe 1	sp. oral taxon 132, 150
Veillonellaceae Genus probe 2	sp. oral taxon 129, 132, 150, 918
Veillonellaceae Genus probe 3	sp. oral taxon 135, 145, 148, 155, 483
Yersinia Genus probe	frederiksenii, pestis, pseudotuberculosis, similis
