# Supplementary material for: Self-reported bovine milk intake is associated with oral microbiota composition
Source: PLoS One. 2018 Mar 21;13(3):e0193504. doi: 10.1371/journal.pone.0193504 (PMC5862454; doi:10.1371/journal.pone.0193504)
Supplement: S3 Table — (PDF) [file pone.0193504.s003.pdf]

S3 Table. Detection of mutans streptococci and lactobacilli in adolescents classified into tertiles based on their reported intake of total milk, non-fermented milk and fermented milk, respectively.

ADOLESCENTS	Low tertile	Middle tertile	High tertile	p-value trend
**NON-FERMENTED MILK (numbers)**	**49**	**58**	**47**	–
**Reported milk intake**, mean (95% CI) of servings/day	0.18 (0.12, 0.24)	1.23 (1.02, 1.44)	2.74 (2.46, 3.02)	<0.001
Caries, DeFS[4], mean (95% CI)	4.4 (2.6, 6.2)	6.4 (4.1, 8.7)	5.0 (2.5, 7.2)	0.406
**Cultivation for viable bacteria, CFU/ml saliva**				
mutans streptococci, median (interquartile range)	1,200 (11,080)	390 (13,180)	0 (5,740)	0.015
lactobacilli, median (interquartile range)	128 (683)	60 (2.215)	28 (375)	0.025
***S. mutans* in tooth biofilm by DNA**				
positive by PCR, %	44.9	48.3	19.6	0.013
abundance by sequencing, mean (95% CI)	1.10 (0.00, 2.44)	0.92 (0.12, 1,72)	0.12 (0.001, 0.24	0.018
***S. mutans* in saliva by DNA**				
positive by PCR, %	65.3	62.1	56.5	0.382
abundance by sequencing, mean (95% CI)	0.06 (0.04, 0.08)	0.09 (0.04, 0.13)	0.06 (0.02, 0.11)	0.100
***S. sobrinus* in tooth biofilm by DNA**				
positive by PCR, %	2.0	1.7	2.2	0.965
***S. sobrinus* in saliva by DNA**				
positive by PCR, %	2.0	3.4	2.2	0.960
**FERMENTED MILK (numbers)**	**45**	**58**	**51**	–
**Reported milk intake**, mean (95% CI) of servings/day	0.04 (0.03, 0.05)	0.26 (0.23, 0.29)	1.17 (0.97, 1.34)	<0.001
Caries, DeFS[4], mean (95% CI)	6.8 (3.7, 10.0)	5.0 (3.3, 7.7)	4.3 (2.6, 6.0)	0.258
**Cultivation for viable bacteria, CFU/ml saliva**				
mutans streptococci, median (interquartile range)	2,500 (27,000)	750 (10,125)	0 (1,040)	<0.001
lactobacilli, median (interquartile range)	100 (1,428)	70 (1,050)	35 (360)	0.068
***S. mutans* in tooth biofilm by DNA**				
positive by PCR, %	44.9	48.3	19.6	0.013
abundance by sequencing, mean (95% CI)[2]	1.80 (0.18, 3.43)	0.24 (0.05, 0.43)	0.27 (0.00, 0.67)	0.004
***S. mutans* in saliva by DNA**				
positive by PCR, %	65.3	62.1	56.5	0.382
abundance by sequencing, mean (95% CI)[2]	0.12 (0.06, 0.17)	0.02 (0.02, 0.08)	0.06 (0.02, 0.09)	0.051
***S. sobrinus* in tooth biofilm by DNA**				
positive by PCR, %	2.0	1.7	2.2	0.965
***S. sobrinus* in saliva by DNA**				
positive by PCR, %	2.0	3.4	2.2	0.960

Means and 95% CI limits adjusted for sex in adolescents and for sex, age and education in adults
