# Supplementary figures and images for: Self-reported bovine milk intake is associated with oral microbiota composition
Source: PLoS One. 2018 Mar 21;13(3):e0193504. doi: 10.1371/journal.pone.0193504 (PMC5862454; doi:10.1371/journal.pone.0193504)

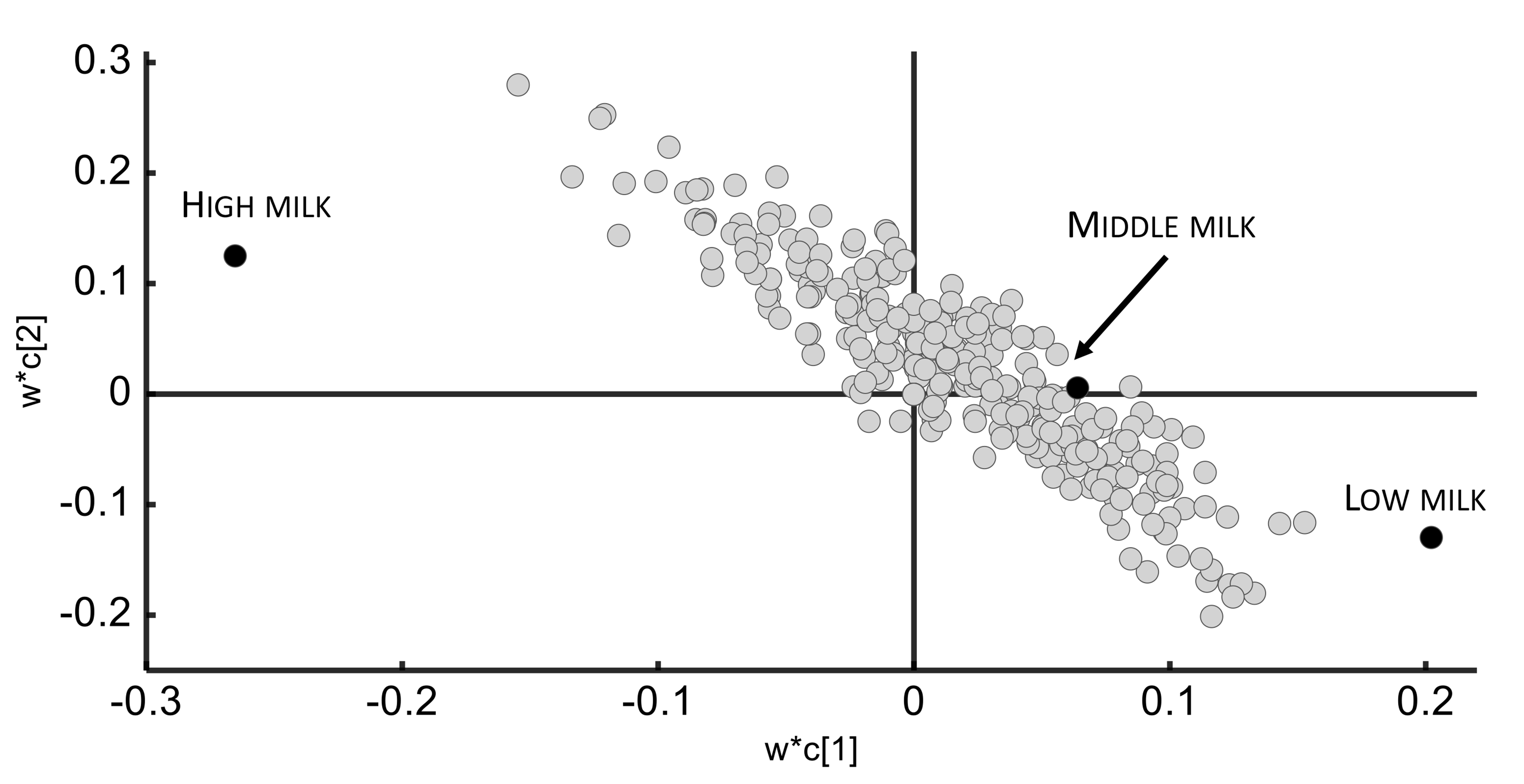

Supplement: S1 Fig — (TIF) [file pone.0193504.s004.tif]

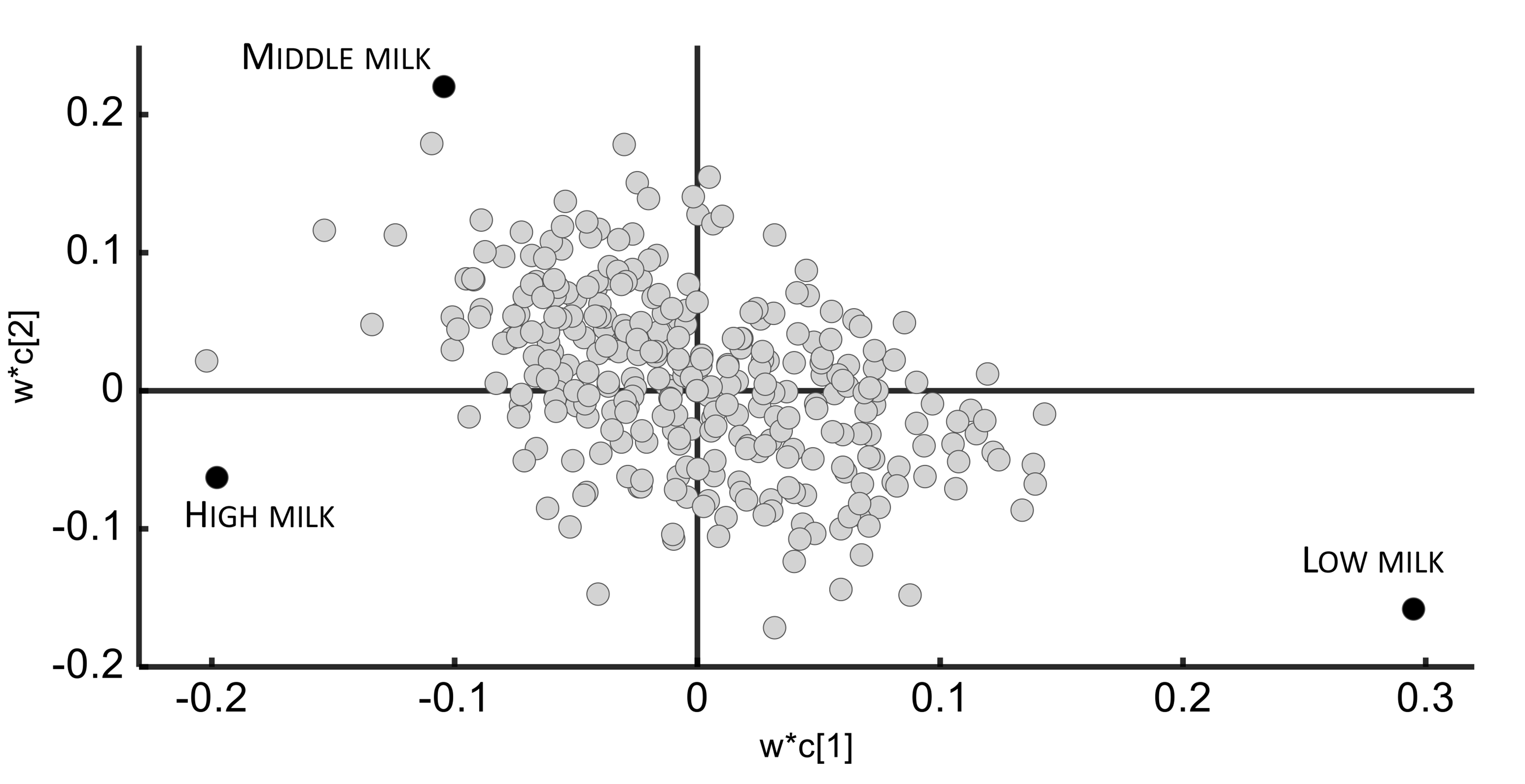

Supplement: S2 Fig — (TIF) [file pone.0193504.s005.tif]
